# *LOC387715/HTRA1* gene polymorphisms and susceptibility to age-related macular degeneration: A HuGE review and meta-analysis

**Published:** 2010-10-05

**Authors:** Yu Tong, Jing Liao, Yuan Zhang, Jing Zhou, Hengyu Zhang, Meng Mao

**Affiliations:** 1 Laboratory of Early Development and Injuries, Center for Research of Child Development and Disease, West China Second University Hospital, Chengdu, China; 2 Department of Pediatrics, Laboratory of Early Development and Injuries, Center for Research of Child Development and Disease, West China Second University Hospital, Sichuan University, Chengdu, China; 3 Department of Science and Technology, Sichuan People's Provincial Hospital, Chengdu, China; 4 Department of Community Health, Wuhou Health Bureau, Chengdu, China; 5 Department of Laboratory Medicine, West China Hospital, Sichuan University, Chengdu, China; 6 Department of Cardiology, West China Hospital, Sichuan University, Chengdu, China

## Abstract

**Purpose:**

To examine the association of age-related macular degeneration (AMD) with HtrA serine peptidase 1 (*HTRA1)* gene rs11200638 G→A polymorphism and *LOC387715/ ARMS2* gene rs10490924 G→T polymorphisms, and to evaluate the magnitude of the gene effect and the possible genetic mode of action.

**Methods:**

We searched the US National Library of Medicine’s PubMed, Embase, OMIM, ISI Web of Science, and CNKI databases in a systematic manner to retrieve all genetic association studies on the *HTRA1* (rs11200638) and *LOC387715/ ARMS2* (rs10490924) gene polymorphisms and AMD. We performed a meta-analysis conducted with Stata software, version 9.0.

**Results:**

Individuals who carried the AA and AG genotypes of *HTRA1* gene rs11200638 G→A polymorphism had 2.243 and 8.669 times the risk of developing AMD, respectively, when compared with those who carry the GG genotype. Individuals carrying the TT and TG genotypes of *LOC387715/ ARMS2* gene rs10490924 G→T polymorphism had 7.512 and 2.353 times the risk of developing AMD, respectively, compared with those who carry GG genotype. These results suggested a “moderate” codominant, multiplicative genetic mode; that is, both *HTRA1* rs11200638 G→A polymorphism and *LOC387715/ARMS2* rs10490924 G→T polymorphism play important roles in the pathogenesis of AMD. We found no evidence of publication bias. Between-study heterogeneity was found in both allele-based analysis and genotype-based analysis.

**Conclusions:**

*HTRA1* rs11200638 G→A polymorphism and *LOC387715/ARMS2* rs10490924 G→T polymorphism play important roles in AMD. Gene-gene and gene-environmental interactions, as well as precise mechanisms underlying common variants in the *HTRA1* gene and *LOC387715/ ARMS2* gene, potentially increase the risk of AMD and need further exploration.

## Introduction

Age-related macular degeneration (AMD) is a neurodegenerative disease that leads to visual impairment and accounts for half of all cases of registered blindness in Western individuals older than 65 years of age [1-14]. There are approximately eight million people in the United States with symptoms of early or intermediate AMD, of whom approximately one million will develop advanced AMD within the next five years [15-17]. AMD is estimated to affect about 50 million people worldwide [18-20], and an increase in aging populations makes AMD a significant public health concern and a major focus of research efforts (National Advisory Council).

AMD is a clinically heterogeneous and genetically complex disease, with multiple environmental and genetic risk factors involved [20-25]. While epidemiological studies have linked cigarette smoking, alcohol consumption, light exposure, diet, drugs, and high blood pressure to the risk of AMD [19,23,26-36], familial aggregation and twin studies [37-43] have suggested that genetic variation may also play an important role in the disease. Although AMD has been reported to be associated with genetic variations in the genes of adenosine-triphosphate (ATP)-binding transporter protein 4 [44-46], apolipoprotein E [47-52], excision-repair cross-complementing group 6 [53], fibulin 5 [54], fibulin 6 [55,56], elongation of very-long-chain fatty acids-like 4 [57-59], factor B/complement component 2 [60], toll-like receptor 4 [61-63], and vascular endothelial growth factor [64], recent genome-wide linkage studies found that genomic regions at chromosomes 1q31–32 and 10q26 may have a bigger role in susceptibility to AMD [65]. The identification of overlapping loci on chromosome 1q by several study groups [66-68] indicates that this locus probably harbors a major AMD-associated gene. Recently, the component factor H (*CFH*) gene on chromosome 1q31 has been revealed as the first major AMD-susceptibility gene, perhaps accounting for about 30%–50% of AMD patients. The *CFH Y402H* variant in the *CFH* gene has also been identified as a causal polymorphism in studies of populations other than those of European and North American origin [30,69-78], and a follow-up meta-analysis [79] has confirmed this association in Western populations. Studies in Japan, however, did not show any associations between *CFH Y402H* polymorphism and AMD [80,81], suggesting that there must be some other loci susceptible for AMD. Several studies have showed that a locus at chromosome 10q26 [82-84] of *CFH* may independently contribute to AMD susceptibility [65,76,82-84]. Three genes identified at chromosome 10q26 and associated with the risk of AMD are Pleckstrin Homology Domain-containing Protein Family A member 1, age-related maculopathy susceptibility 2 (*LOC387715/age-related maculopathy susceptibility 2 [ARMS2]*), and high-temperature requirement factor A1 (*HTRA1/PRSS11*) [65,76,82-84]. Thus, AMD appears to be a product of the interaction between multiple loci of susceptibility rather than a collection of single-gene disorders. However, the number of loci involved, the degree of attributable risk conferred, and the interactions between various loci remain obscure.

The *HTRA1* gene spans a 53,366-base region on chromosome 10q26 (124211047–124264413, Gene ID: 5654); it encodes a member of a family of serine proteases expressed in both mouse and human retinas [85,86], and its expression in human fibroblasts increases with aging [87]. HTRA1 appears to regulate the degradation of extracellular matrix proteoglycans. This activity has been considered to facilitate access of other degradative matrix enzymes, such as collagenases and matrix metalloproteinases, to their substrates [88]. Overexpression of HTRA1 alters the integrity of Bruch’s membrane, favoring the invasion of choroid capillaries across the extracellular matrix, as occurs in wet AMD. HTRA1 also binds and inhibits transforming growth factor-β (TGF-β), an important regulator of extracellular matrix deposition and angiogenesis [89]. During the years 2006 to 2008, several studies were conducted to investigate the association between HTRA1 gene polymorphisms and AMD. A single-nucleotide polymorphism (rs11200638) in the promoter region of the HTRA1 gene was found to be significantly associated with susceptibility to AMD in studies of Caucasian populations in the US [90-97], Central Europe [98], France [99], and the UK [100]; of East Asian populations in China [101-104] and Japan [105-107]; and of Indian populations in India [108]. Another putative AMD-susceptibility gene, *LOC387715/ARMS2*, has recently been identified. *LOC387715/ARMS2* encodes a deduced 107–amino acid protein with nine predicted phosphorylation sites and a molecular mass of 12 kDa. Real-time (RT)-PCR analysis demonstrated that *LOC387715/ARMS2* transcripts were expressed in the retina and in a variety of other tissues and cell lines. Transfection experiments in mammalian cells localized the protein to the mitochondrial outer membrane [95]. Up to now, the biologic characterization of this gene has been limited. However, Rivera et al. [109] concluded that the A69S single-nucleotide polymorphism (rs10490924) in exon 1 of the *LOC387715/ARMS2* gene was the most likely susceptibility allele of AMD. Since an individual study may not have sufficient statistical robustness to confirm the association between *HTRA1* and *LOC387715/ARMS2* gene polymorphisms and AMD, we considered that a meta-analysis that combined data from all published studies would provide a more accurate estimate of the extent of association, leading to less risk of false-positive results [110]. Thus, we systematically pooled the results of all available population-based association studies of the *HTRA1* rs11200638 G→A polymorphism, the *LOC387715/ARMS2* rs10490924 G→T polymorphism, and AMD. We attempted to estimate the strength of the genetic association with AMD, as well as the genetic mode of action, and to gauge the extent of heterogeneity in the strength of the associations among different studies.

## Methods

### Search strategy and inclusion criteria

We searched the US National Library of Medicine’s PubMed, Embase, OMIM, ISI Web of Science, and Chinese National Knowledge Infrastructure (CNKI) databases in a systematic manner to retrieve all genetic association studies on the *HTRA1* (rs11200638) and *LOC387715/ARMS2* (rs10490924) polymorphisms and AMD published before April 2008. The search strategy was based on a combination of the terms (HtrA serine peptidase 1 or *HTRA1*), (*age-related maculopathy susceptibility 2 or LOC387715*), and (age-related macular degeneration or AMD). The references of all computer-identified publications were searched for additional studies, and the PubMed option ‘‘Related Articles’’ was also used to search for potentially relevant papers. Searches were performed by two independent reviewers (B.Z. and J.Y.). We included all published articles regardless the language of publication.

Studies were included if they met the following criteria: 1) The study reported original data from case-control or cohort studies. 2) The alleles and genotypes for the *HTRA1* polymorphism (rs11200638), respectively, were A and G and AA, AG, and GG. 3) The alleles and genotypes for the *LOC387715/ARMS2* polymorphism (rs10490924), respectively, were G and T and GG, GT, and TT. 4) The numbers of subjects possessing each allele and genotype in the AMD and control groups were available. 5) In the case of multiple publications from the same study group, the most complete and recent results were used. We set no restriction on the source of controls (general population, clinic, or hospital). For those studies where AMD was graded (e.g., drusen, pigment abnormalities in retinal pigment epithelium [RPE], geographic atrophy, and choroidal neovascularization [CNV]), the gradings were combined into a single AMD group.

### Data extraction

Data were extracted independently by two investigators (B.Z. and J.Y.), who used recommended guidelines to report on meta-analyses of observational studies [111]. The following data were extracted from the eligible studies: authors, journal title and year of publication, country of origin, selection and characteristics of cases and controls, demographic data, ethnicity of the study population (e.g., Caucasian or East Asian), numbers of eligible and genotyped cases and controls, and genotype distributions in cases, controls, and available subgroups. Furthermore, we examined whether matching had been used; whether there was specific mention of blinding of the genotyping personnel to the clinical status of subjects; whether the genotyping method used had been validated; and whether genotype frequencies in control groups conformed to the Hardy–Weinberg equilibrium (HWE). Any disagreement was adjudicated by a third author (R.L.).

### Statistical analysis

We used the odds ratio as the metric of choice and this was estimated for each study. To explore the possible association between *HTRA1* and *LOC387715/ARMS2* polymorphisms and AMD, and to avoid excessive comparisons, we calculated the odds ratio by two methods: allele comparison (the A allele versus the G allele in the *HTRA1* rs11200638 G→A polymorphism), and comparing the risk-variant homozygotes and heterozygotes with wild homozygotes (i.e., AA versus GG [*OR*_1_] and AG versus GG [*OR*_2_] in the *HTRA1* rs11200638 G→A polymorphism). We estimated and characterized the prevalence of the risk allele with only the data from controls. When we analyzed genotype data in the meta-analysis, zero cell counts were assigned a fixed value (typically 0.5). In addition, we calculated the population attributable risk (PAR) of the risk allele according to the Chang et al. [112] method.

We first compared the alleles for cases and controls to detect overall differences and genetic association. Allele frequencies were computed for studies reporting only genotypic data. Pooled odds ratios were computed two times: by the fixed effects model of Mantel and Haenszel [113], and by the random effects model of DerSimonian and Laird [114]. Random effects incorporated an estimate of between-study variance and provided wider confidence intervals when the results of the constituent studies differed. The random effects model was more appropriate when heterogeneity was present [115]. Unless otherwise stated, the random effects estimates reported here were calculated by the DerSimonian and Laird model.

Our primary genetic analysis of the *HTRA1* rs11200638 G→A polymorphism, the *LOC387715/ARMS2* rs10490924 G-to -T polymorphism, and AMD was based on the comparisons between risk-variant homozygotes and heterozygotes versus wild homozygotes so that the strength of the genetic association and the genetic mode of action could be identified exactly. Once an overall gene effect was confirmed, the genotype effects and genetic model were estimated by using the genetic model-free approach suggested by Minelli et al. [116], in which no assumptions about genetic models are required. A multivariate meta-analysis employing the Bayesian method [116] was used to calculate *OR*_1_ and *OR*_2_. The logarithm (log) odds ratios were modeled on the basis of both between- and within-study variations. A stochastic parameter lambda (λ), equal to the ratio of log *OR*_2_ and log *OR*_1_, was also computed [115]. The parameter λ suggested the genetic mode of action; specifically, the model is a recessive model if λ=0, a codominant model if λ=0.5, a dominant model if λ=1, and homozygous or heterosis model if λ<0 or λ>1.

We examined the deviations from the HWE in control populations for each study by using the exact method [117]. For all the analyses, we compared results between inclusion and exclusion of studies in Hardy–Weinberg (HW) disequilibrium. In addition, all studies were included regardless of HWE and provided a revision of the degree of HW disequilibrium by using the inbreeding coefficient (*F*) suggested by Trikalinos et al. [118]. In brief, data in the control group were used to assess the *F* value for each study. Predicted genotype frequencies were estimated and then used to replace the observed frequencies in the summary analysis of magnitude and the genetic model.

In sensitivity analysis, we estimated between-study heterogeneity across all eligible comparisons using Cochran’s *Q* statistic [115]. We also reported the *I*^2^ statistic, which describes the percentage of variability in point estimates due to sample heterogeneity rather than sampling error [119,120], and can quantify heterogeneity irrespective of the number of studies [120,121]. *I*^2^ values larger than 75% were considered to represent a “notable” heterogeneity [120,121]. Publication bias among studies was assessed by funnel plots [122] and cumulative meta-analysis [123]. In the analysis of subgroups, we estimated odds ratios according to racial descent (Caucasians versus East Asians) and AMD type (wet AMD and other subtype or combined AMD).

All analyses were conducted with Stata software, version 9.0 (StataCorp, 2005) [124], using the *meta, metan, metabias, metacum, and metareg* commands, except the Bayesian method of genotype-based analysis. We fitted the Bayesian models by using Markov chain Monte Carlo methods with a Bayesian framework and performed our inferences using WinBUGS 1.4.3 (Imperial College School of Medicine at St Mary's, London 2003) [125], taking advantage of its flexibility as well as its ability to incorporate full uncertainty across all unknown parameters. Bayesian analyses yielded credible intervals rather than confidence intervals; a 95% credible interval (*CrI*) describes a range in which it is probable that an unknown quantity lies within this interval. A “burn-in” of 10,000 iterations is performed for models, followed by 50,000 iterations for parameter estimates. A p value less than 0.05 was considered statistically significant.

## Results

### Eligible studies

A total of 29 studies were identified based on our search strategies, of which 13 studies [95-106,108] were eligible for inclusion in this meta-analysis; all of these were written in English. One [101] did not report genotype information in their paper, but online supporting materials provided the data. Two of the studies [100,106] did not have genotypic data, but the authors kindly sent the supplementary information to us. Sixteen studies were ineligible for the following reasons: six were reviews [22,24,126-129], six did not study the association between the *HTRA1* rs11200638 G→A polymorphism and AMD [92-94,130-132], two [90,91] were duplicated reports of the most recent and comprehensive one [97], and one did not have genotype data [107].

Detailed characteristics of the 13 included studies on the association between *HTRA1* rs11200638 G→A polymorphism and AMD are presented in Table 1. Among them, six studies related to Caucasian subjects, six to East Asians, and one to Indians. The average age of subjects ranged from 64.0 years to 81.2 years for cases and from 64.0 years to 77.4 years for controls. Characteristics of the 13 included studies on the association between *LOC387715/ARMS2* rs10490924 G→T polymorphism and AMD are presented in Table 2. Among them, 14 studies related to Caucasians, three to East Asians, and one to Indians. The average age ranged from 60 to 79 years for cases and from 60 to 77 years for controls. All of the eligible studies had case-control designs. Cases in the studies were recruited from hospital patients and controls were mainly healthy populations recruited from the hospital or community and unrelated to cases.

**Table 1:** Characteristics of case-control studies included in a meta-analysis of the association between the *HTRA1* gene polymorphisms and AMD

**Ref**	**Year**	**Region, country study was conducted**	**Ethnicity**	**Study design**	**Sex composition in cases (% males)**	**Mean age (years)**		**Cases**	**Controls**	**Number of eligible subjects**	
**Cases**	**Controls**	**Cases**	**Controls**
[101]	2006	China	East Asian	Case-control	68	74.9	74.2	Wet AMD	Age matched controls without AMD, confirmed by full ophthalmologic examination	96	130
[98]	2007	Austria	Caucasian	Case-control	35.5	78	77.4	Exudative AMD in AMD level 4	Caucasians without AMD on the base of a detailed eye examination and fundus examination	242	157
[102]	2007	China	East Asian	Case-control	45.1	64.0/	64	Drusen, and wet AMD	Without any AMD, confirmed by a normal eye examination	164	106
[105]	2007	Japan	East Asian	Case-control	72.4	71.9	67.9	AMD, combined	Without AMD and unrelated to cases, confirmed by full ophthalmologic examination	123	133
[106]	2007	Japan	East Asian	Case-control	79.5	75.7	71.2	Wet AMD	Hospital-based controls without retinal diseases and AMD on the base of full ophthalmologic examination	73	94
[95]	2007	USA	Caucasian	Case-control	NR	>68.0	>68.0	AMD, combined	Without AMD on the base of full ophthalmologic examination	535	288
[99]	2007	France	Caucasian	Case-control	NR	>65.0	>65.0	Exudative AMD	Without any type of drusen, geographic atrophy, or exudative AMD.	200	116
[96]	2007	USA	Caucasian	Case-control	NR	71.3	72.8	Wet AMD	Without AMD on the base of full ophthalmologic examination	134	134
[100]	2007	UK	Caucasian	Case-control	40.6	>65.0	>65.0	Wet AMD	Without AMD on the base of full ophthalmologic examination	401	266
[97]	2008	USA	Caucasian	Case-control	49.0/ 52.5/ 38.0/ 44.4	81.2/ 78.9/ 81.0/ 78.3	74	bilateral wet AMD, unilateral wet AMD, bilateral GA, and unilateral GA.	Without any type of drusen, GA, AMD, and RPE	776	294
[103]	2008	China	East Asian	Case-control	54	71.2	71.5	Dry and wet AMD	Age and sex matched controls without any visual impairment, excluded a family history of AMD and any type of drusen, geographic atrophy, CNV, or other retinal disorder in either eye.	95	90
[104]	2008	China	East Asian	Case-control	54	75.5	73.3	Exudative AMD	Without any AMD and any other major eye diseases	163	183
[108]	2008	India	Indian Asian	Case-control	NR	68.8	64.4	AMD, combined	Ethnic matched controls, without a family history of AMD or any other ocular or systemic diseases	250	250

**Table 2:** Characteristics of case-control studies included in a meta-analysis of the association between the *LOC387715* gene polymorphisms and AMD

**Ref**	**Year**	**Region, country study was conducted**	**Ethnicity**	**Study design composition in cases (% males)**	**Sex**	**Mean age (years)**		**Cases**	**Controls**	**Number of eligible subjects**	
**Cases**	**Controls**	**Cases**	**Controls**
[106]	2007	Japan	East Asian	Case-control	79.5	75.7	71.2	Wet AMD	Hospital-based controls without retinal diseases and AMD on the base of full ophthalmologic examination	73	94
[95]	2007	USA	Caucasian	Case-control	NR	>68.0	>68.0	Wet AMD+Dry AMD	Without AMD on the base of full ophthalmologic examination	431	280
[99]	2007	France	Caucasian	Case-control	NR	>65.0	>65.0	Wet AMD	Without any type of drusen, geographic atrophy, or exudative AMD.	118	116
[96]	2007	USA	Caucasian	Case-control	NR	71.3	72.8	Wet AMD	Without AMD on the base of full ophthalmologic examination	134	134
[100]	2007	UK	Caucasian	Case-control	40.6	>65.0	>65.0	Wet AMD	Without AMD on the base of full ophthalmologic examination	401	266
[108]	2008	India	Indian Asian	Case-control	NR	68.8	64.4	Wet AMD+Dry AMD	Ethnic matched controls, without a family history of AMD or any other ocular or systemic diseases	193	203
[3]	2008	China	East Asian	Case-control	58.7	66	66.1	Wet AMD	Without any AMD and any other major eye diseases aside from mild age-related cataracts	121	132
[84]	2005	Germany	Caucasian	Case-control	NR	NR	NR	Wet AMD+Dry AMD	Without any AMD and any other major eye diseases	759	594
[84]	2005	Germany	Caucasian	Case-control	35.1	75.01	68.25	Wet AMD+Dry AMD	Unrelated controls without any AMD and any other major eye diseases	361	328
[133]	2006	USA	Caucasian	Case-control	42	79.5	76.5	Wet AMD+Dry AMD	Without AMD on the base of full ophthalmologic examination	693	172
[133]	2006	USA	Mixed	Case-control	44	73.2	70.3	Wet AMD+Dry AMD	Without AMD on the base of full ophthalmologic examination	120	995
[56]	2007	Russia	Caucasian	Case-control	27.7	72.6	71.1	Wet AMD+Dry AMD	Free of macular changes	155	151
??	2007	Japan	East Asian	Case-control	70.5	73.4	73.6	Wet AMD	Without any AMD	95	99
??	2007	USA	Caucasian	Nested case-control	35.2	60.1	60.2	Wet AMD+Dry AMD	Within 1 year of the same age with cases, and underwent eye examination in the past 2 years	445	1041
[128]	2007	USA	Caucasian	Case-control	42.6	79	72	Wet AMD+Dry AMD	AMD free controls	399	329
[13]	2007	Australia	Caucasian	Cohort	39.9	75.6	74.9	Wet AMD+Dry AMD	AMD free controls	278	557
[93]	2007	USA	Caucasian	Case-control	NR	NR	NR	Wet AMD	Without any AMD	87	232
[83]	2008	USA	Caucasian	Case-control	39.6	79.1	72.9	Wet AMD+Dry AMD	Without AMD on the base of full ophthalmologic examination	164	155

### Allele comparison

Data from the control groups were used to calculate the summary allele frequency. The frequency of the risk allele A in the *HTRA1* rs11200638 G→A polymorphism among controls was 32.33% (95% confidence interval [*CI*]: 26.29, 38.38), and was significantly higher in Asians than in Caucasians (40.11% [95% *CI*: 35.11, 45.12] versus 23.25% [95% *CI*: 18.41, 28.09], p=0.0001). The frequency of the risk allele T in the *LOC387715/ARMS2* rs10490924 G→T polymorphism among controls was 25.17% (95% *CI*: 17.33, 33.00), and was also significantly higher in Asians than in Caucasians (38.67% [95% *CI*: 34.63, 42.71] versus 21.62% [95% *CI*: 17.41, 28.83], p=0.0000178).

**Figure 1:**
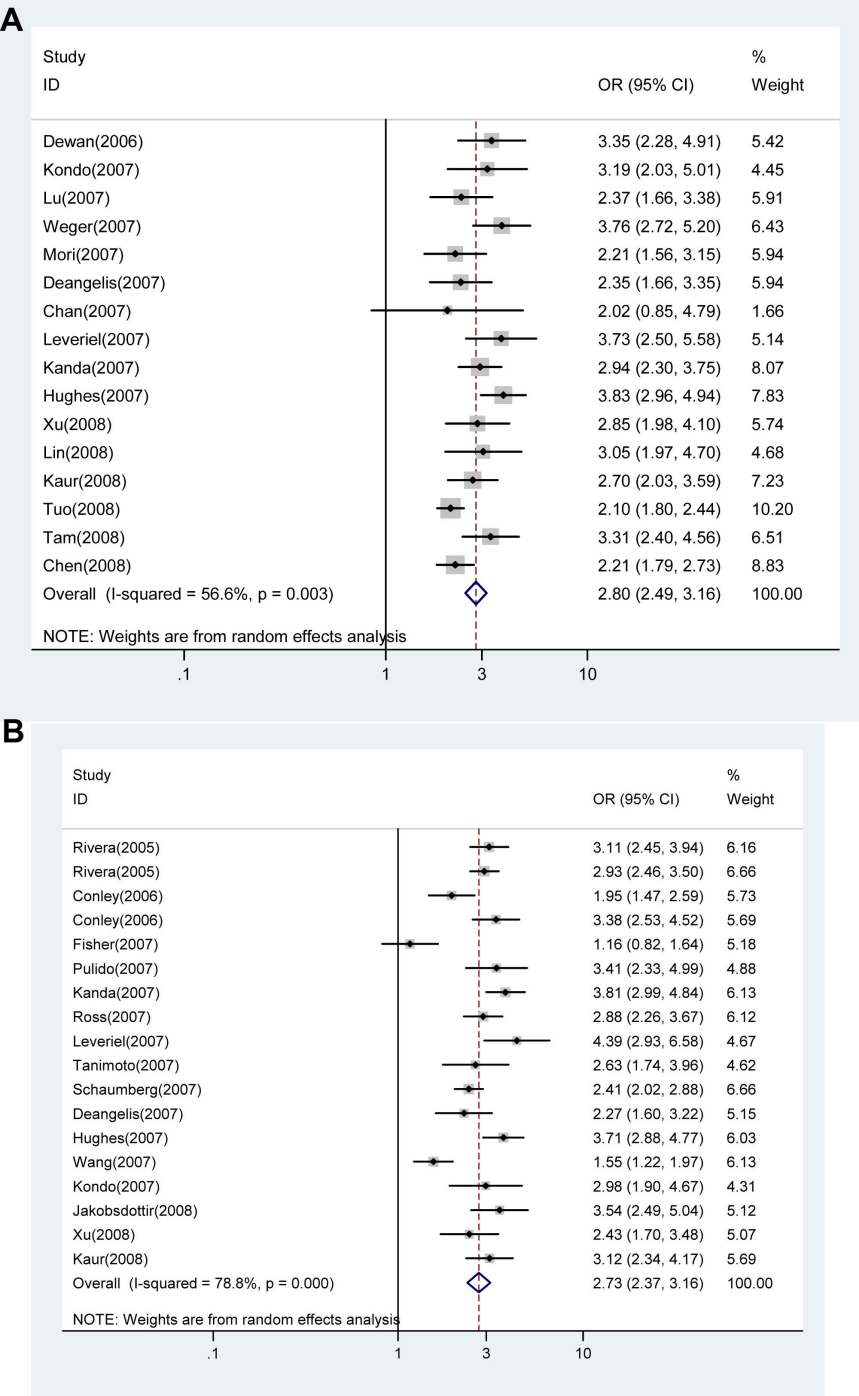
Random-effects meta-analysis of allele (A versus G) of the HTRA1 gene rs11200638 G→A polymorphism and age related macular degeneration (AMD). **A**: Results from random-effects meta-analysis of allele (A versus G) of the *HTRA1* gene rs11200638 G→A polymorphism and AMD. **B**: Results of the random-effects meta-analysis of the allele (T versus G) of the *LOC387715/ARMS2* gene rs10490924 G→T polymorphism and AMD. Reference numbers are given in parentheses. For study details, see Table 1 and Table 2.

**Figure 2:**
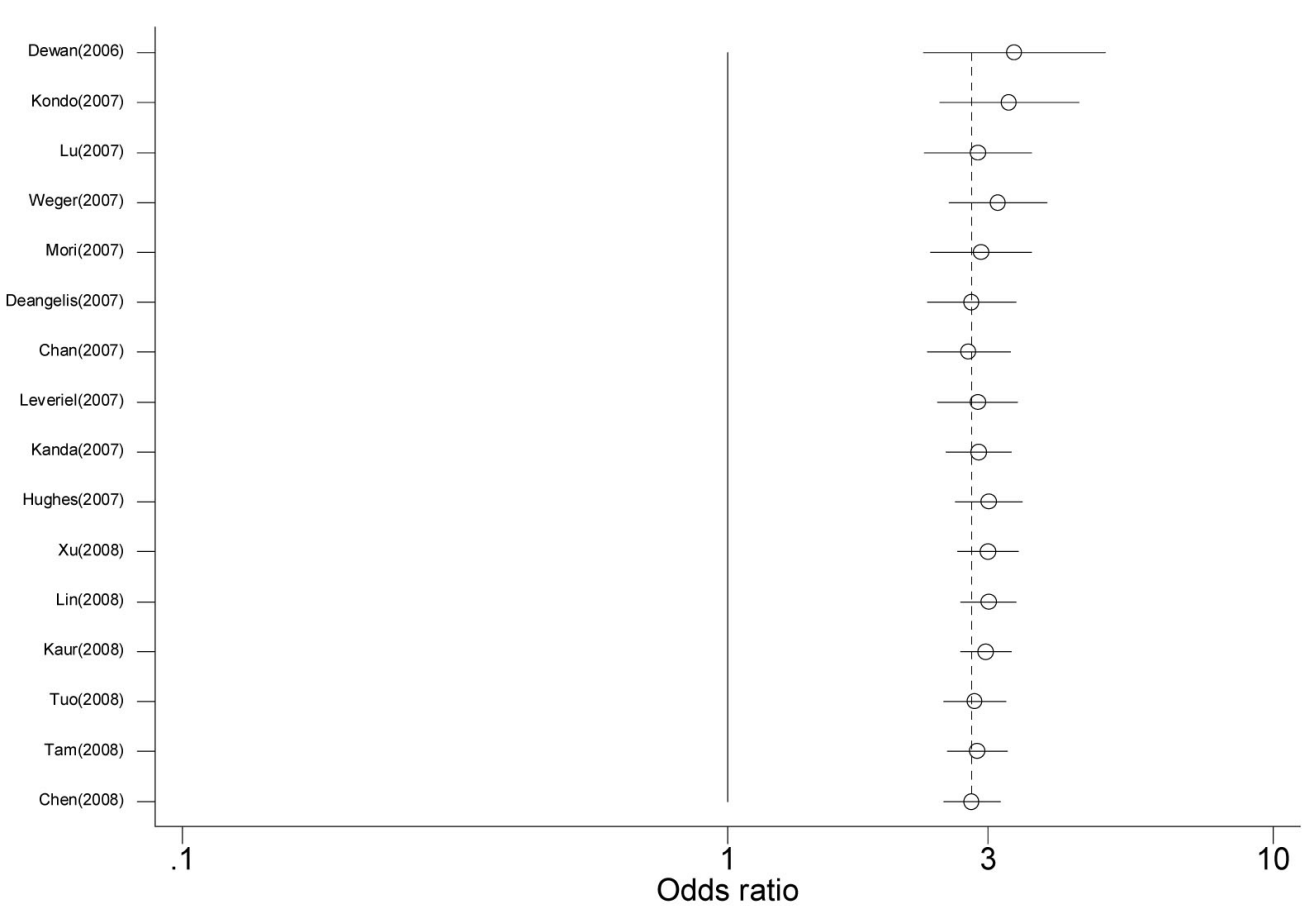
Cumulative random-effects meta-analysis of allele (A versus G) of the *HTRA1* gene rs11200638 G→A polymorphism and age related macular degeneration (AMD). For study details, see Table 3.

All of the 13 studies were included to evaluate the association between the *HTRA1* rs11200638 G→A polymorphism and AMD [95-106,108]. As shown in Figure 1A, individuals with the A allele experienced a 2.80-fold increased risk of AMD when compared to individuals with the G allele (random effect *OR*=2.910, 95% *CI*: 2.552, 3.318; *Q*=25.769, p=0.012, *I*^2^=53.4%). The magnitude of the effect was similar for Asians (random effect *OR*=2.841, 95% *CI*: 2.482, 3.252) and Caucasians (random effect *OR*=2.981, 95% *CI*: 2.357, 3.370). However, there was significantly greater between-study heterogeneity among Caucasians (*Q*=20.128, p=0.001, *I*^2^=75.2%) than Asians (*Q*=5.636, p=0.465, *I*^2^=0.0%). Excluding and adjusting two studies [96,97] with Hardy–Weinberg equilibrium did not change the results (data not shown). After appropriately carrying out a set of prespecified subgroups [97], a low level of between-study heterogeneity was found (random effect *OR*=3.043, 95% *CI*: 2.725, 3.397; *Q*=14.318, p=0.216, *I*^2^=23.2%). We did not find any evidence of publication bias in the eligible studies (corrected Begg’s test *z*=0.43, corrected p=0.669). Figure 2 shows the cumulative meta-analysis results; they remained significant and were consistent over time.

**Figure 3:**
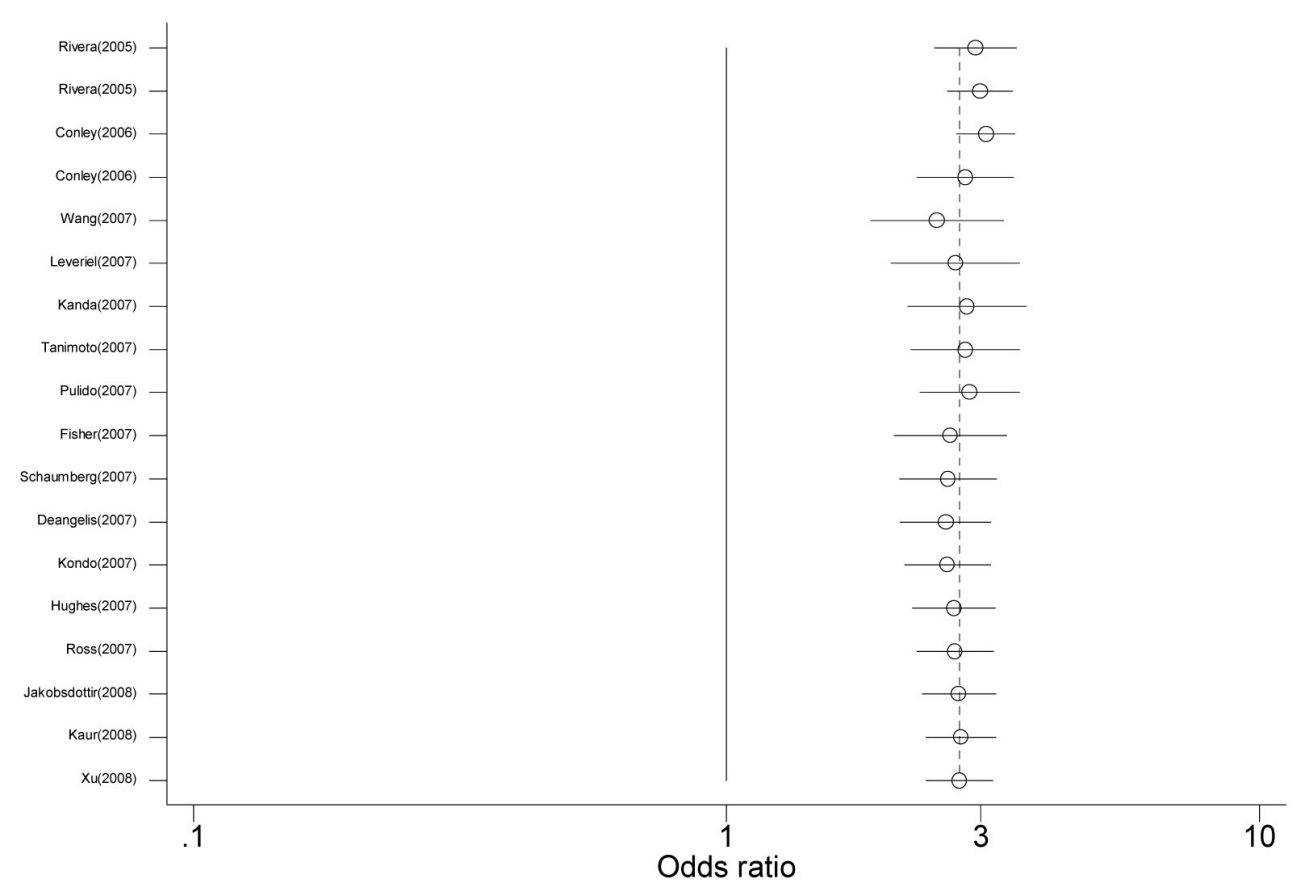
Cumulative random-effects meta-analysis of allele (G versus T) of the *LOC387715/ ARMS2* gene rs10490924 G→T polymorphism and age related macular degeneration (AMD). For study details, see Table 4.

The association between the *LOC387715/ ARMS2* rs10490924 G→T polymorphism and AMD was also evaluated. As shown in Figure 1B, individuals with the T allele had a 2.734 fold increased risk of AMD when compared to individuals with the G allele (random effect *OR*=2.734, 95% *CI*: 2.366, 3.158; *Q*=80.195, p=0.000, *I*^2^=78.8%). The magnitude of the effect was similar between Asians (random effect *OR*=2.692, 95% *CI*: 2.086, 3.315) and Caucasians (random effect *OR*=2.794, 95% *CI*: 2.333, 3.346). There was also a significant difference between-study heterogeneity among Caucasians (*Q*=73.265, p=0.000, *I*^2^=83.6%) as opposed to Asians (*Q*=0.481, p=0.786, *I*^2^=0.0%). Figure 3 shows the cumulative meta-analysis results; they remained significant and were consistent over time.

### Genotype comparison

The genotype frequency of the *HTRA1* rs11200638 G→A polymorphism between case and control groups is presented in Table 3. The genotype effects for AA versus GG (*OR*_1_) and AG versus GG (*OR*_2_) were calculated for each study. The genotype frequency of the *LOC387715/ARMS2* rs10490924 G→T polymorphism between the case and control groups is presented in Table 4. The genotype effects for TT versus GG (*OR*_1_) and TG versus GG (*OR*_2_) were calculated for each study.

**Table 3:** The association between the *HTRA1* gene polymorphisms and AMD—- Allele and genotype frequencies of case-control studies included in a meta-analysis

**Ref**	**Year**	**Genotype distribution**										**AA / GG**		**AG / GG**		**A / G**	
**cases**					**controls**				
**N**	**AA**	**AG**	**GG**	**P value for HWE**	**N**	**AA**	**AG**	**GG**	**P value for HWE**	**OR1**	**95% CI**	**OR2**	**95% CI**	**OR**	**95% CI**
[101]	2006	96	44	40	18	0.266	130	14	59	57	0.976	9.952	4.465~22.184	2.147	1.104~4.174	3.626	2.450~5.368
[98]	2007	242	67	108	67	0.247	157	8	50	99	0.877	12.375	5.583~27.432	3.192	2.022~5.039	3.723	2.693~5.158
[102]	2007	164	68	77	19	0.924	106	15	63	28	0.104	6.681	2.980~14.979	1.801	0.921~3.523	2.37	1.664~3.375
[105]	2007	123	45	55	26	0.488	133	22	57	54	0.582	4.248	2.127~8.487	2.004	1.103~3.640	2.231	1.566~3.178
[106]	2007	73	29	39	5	0.239	94	16	40	38	0.627	13.775	4.520~41.984	7.41	2.642~20.786	3.189	2.029~5.013
[95]	2007	457	102	183	172	<0.001	280	11	90	179	0.997	9.65	5.006~18.601	2.112	1.525~2.937	2.937	2.299~3.753
[99]	2007	118	32	57	29	0.937	116	5	41	70	0.948	15.448	5.476~43.582	3.356	1.860~6.055	3.734	2.498~5.583
[96]*	2007	134	43	54	37	0.0837	134	21	43	70	0.0111	3.874	2.009~7.469	2.376	1.350~4.180	2.358	1.657~3.347
[100]	2007	401	106	172	123	0.019	266	6	91	169	0.296	24.274	10.327~57.057	2.597	1.841~3.664	3.826	2.963~4.942
[97]*	2008	776	131	400	245	0.327	294	10	128	156	0.0282	8.341	4.253~16.360	1.99	1.500~2.640	2.042	1.652~2.525
[103]	2008	95	53	33	9	0.53	90	19	47	24	0.903	7.439	2.940~18.819	1.872	0.772~4.541	3.046	1.973~4.703
[104]	2008	163	94	51	18	0.0379	183	38	90	55	0.994	7.559	3.398~14.509	1.732	0.919~3.262	3.31	2.403~4.559
[108]	2008	229	90	89	50	0.0111	184	21	85	78	0.956	6.686	3.695~12.098	1.633	1.028~2.595	2.701	2.033~3.589
Total		3071					2167										

* Hardy–Weinberg disequilibrium in case and/or control group

**Table 4:** The association between the *LOC387715* gene polymorphisms and AMD—Allele and genotype frequencies of case-control studies included in a meta-analysis

**Ref**	**Year**	**Genotype distribution**										**TT / GG**		**TG / GG**		**T / G**	
**cases**					**controls**				
**N**	**TT**	**TG**	**GG**	**P value for HWE**	**N**	**TT**	**TG**	**GG**	**P value for HWE**	**OR1**	**95% CI**	**OR2**	**95% CI**	**OR**	**95% CI**
[106]	2007	73	27	40	6	0.25	94	15	41	38	0.783	11.4	3.920~33.155	6.179	2.354~16.217	2.979	1.901~4.668
[95]	2007	431	133	180	118	<0.001	280	12	99	169	0.992	15.875	8.405~29.979	2.604	1.854~3.658	3.809	2.995~4.845
[99]	2008	118	37	55	26	0.811	116	5	40	71	0.978	20.208	7.169~56.962	3.755	2.048~6.886	4.388	2.928~6.576
[96]	2008	134	45	51	38	0.0234	134	22	44	68	0.013	3.66	1.918~6.985	2.074	1.178~3.653	2.271	1.600~3.223
[100]	2005	401	111	170	120	0.00992	266	10	89	167	0.908	15.448	7.761~30.746	2.658	1.878~3.763	3.71	2.884~4.774
[108]	2005	193	81	77	35	0.101	203	25	89	89	0.932	8.239	4.544~14.937	2.2	1.340~3.613	3.123	2.336~4.175
[3]	2006	121	54	49	18	0.472	132	22	70	40	0.651	5.455	2.589~11.491	1.556	0.800~3.026	2.43	1.697~3.480
[84]	2006	759	142	349	268	0.327	594	27	179	388	0.558	7.614	4.904~11.822	2.823	2.225~3.582	2.932	2.459~3.495
[84]	2007	361	88	156	117	0.0471	328	16	109	203	0.962	9.543	5.347~17.030	2.483	1.778~3.468	3.109	2.453~3.940
[133]	2007	693	135	341	217	0.999	172	4	57	111	0.567	17.264	6.223~47.893	3.06	2.131~4.395	1.949	1.466~2.592
[133]	2007	120	18	49	53	0.501	995	43	351	601	0.654	4.747	2.559~8.804	1.583	1.050~2.386	3.384	2.532~4.523
[56]	2007	155	16	66	73	0.982	151	10	66	75	0.669	1.644	0.700~3.859	1.027	0.643~1.643	1.161	0.821~1.641
??	2007	95	39	34	22	0.0398	99	10	50	39	0.58	6.941	2.898~16.491	1.205	0.610~2.380	2.626	1.742~3.958
??	2007	445	68	182	195	0.0694	1041	41	308	692	0.661	5.886	3.872~8.948	2.097	1.645~2.673	2.412	2.023~2.876
[128]	2007	399	69	182	148	0.601	329	12	100	217	0.994	8.431	4.412~16.112	2.669	1.935~3.679	2.883	2.265~3.670
[13]	2007	278	14	120	144	0.216	557	16	179	362	0.547	2.2	1.047~4.623	1.685	1.247~2.278	1.552	1.221~1.974
[93]	2007	87	19	38	30	0.578	232	13	60	159	0.092	7.746	3.459~17.346	3.357	1.911~5.896	3.409	2.331~4.986
[83]	2008	164	40	74	50	0.483	155	10	42	103	0.108	8.24	3.812~17.813	3.63	2.185~6.029	3.54	2.488~5.038
Total		5027					5878										

In our primary analysis, multivariate meta-analysis was conducted to estimate the pooled risk and there was a significantly increased risk of AMD among individuals with both homozygous variant AA genotype (Bayesian random effect *OR*_1_=8.469, 95% *CrI*: 6.766, 10.710) and heterozygous variant AG genotype (Bayesian random effect *OR*_2_=2.243, 95% *CrI*: 1.969, 2.559) of the *HTRA1* rs11200638 G→A polymorphism. A moderate level of between-study heterogeneity (*Q*=19.201, p=0.084, *I*^2^=37.5%) was found for the homozygous AA genotype and no between-study heterogeneity (*Q*=13.951, p=0.304, *I*^2^=14.0%) was found for the heterozygous AG genotype. The estimated parameter λ was 0.378 (95% *CrI*: 0.329, 0.428), which suggested a moderate codominant genetic mode of action. When we removed the two studies [96,97] with HW disequilibrium, similar results appeared with the pooled *OR*_1_, *OR*_2_, and λ of 9.257 (95% *CrI*: 7.267, 11.910), 2.334 (95% *CrI*: 2.012, 2.706), and 0.380 (95% *CrI*: 0.327, 0.435), respectively; however, no significant between-study heterogeneity was found for either the homozygous AA genotype (*Q*=13.898, p=0.178, *I*^2^=28.0%) or the heterozygous AG genotype (*Q*=13.041, p=0.221, *I*^2^=23.3%). The pooled estimates also remained similar after adjusting HW disequilibrium by coefficient *F* (*OR*_1_=9.065 [95% *CrI*: 7.397, 11.180], *OR*_2_=2.306 [95% *CrI*: 2.039, 2.607], and λ=0.379 [95% *CrI*: 0.332, 0.427]).

Multivariate meta-analysis also showed that there was a significantly increased risk of AMD among individuals with both the homozygous variant TT genotype (Bayesian random effect *OR*_1_=7.512, 95% *CrI*\: 5.703, 9.659) and heterozygous variant TG genotype (Bayesian random effect *OR*_2_=2.353, 95% *CrI*: 2.072, 2.665) of the *LOC387715/ ARMS2* rs10490924 G→T polymorphism. The estimated parameter for λ was 0.426 (95% *CrI*: 0.387, 0.467), which suggested a moderate codominant genetic mode of action.

For the *HTRA1* rs11200638 G→A polymorphism, stratification by ethnicity indicated a considerable variation in the size of effects between Asian populations (Bayesian random effect *OR*_1_=7.100, 95% *CrI*: 5.325, 9.494; Bayesian random effect *OR*_2_=2.009, 95% *CrI*: 1.625, 2.511; λ=0.356, 95% *CrI*: 0.267, 0.442) and Caucasian populations (Bayesian random effect *OR*_1_=10.130, 95% *CrI*: 6.323, 0.574; Bayesian random effect *OR*_2_=2.347, 95% *CrI*: 1.918, 2.910; λ=0.368, 95% *CrI*: 0.307, 0.434). A moderate degree of between-study heterogeneity was found for the AA homozygous genotype among both Asians (*Q*=13.978, p=0.030, *I*^2^=57.1%) and Caucasians (*Q*=13.203, p=0.022, *I*^2^=62.1%), but no significant between-study heterogeneity was found for the AG homozygous genotype among either population (Asians: *Q*=7.309, p=0.293, *I*^2^=17.93%; Caucasians: *Q*=5.165, p=0.396, *I*^2^=3.2%). For the *LOC387715/ ARMS2* rs10490924 G→T polymorphism, a moderate level of between-study heterogeneity was found for the TT homozygous genotype among Caucasians (*Q*=45.035, p=0.000, *I*^2^=73.8%) and for the TG heterozygous genotype among both Asians (*Q*=7.783, p=0.020, *I*^2^=74.5%) and Caucasians (*Q*=29.790, p=0.003, *I*^2^=59.7%); however, no significant degree of between-study heterogeneity was found for the TT homozygous genotype among Asians (*Q*=1.232, p=0.54, *I*^2^=0.0%).

Results of metaregression analysis indicated that classification of AMD (wet AMD versus combined AMD) was significantly associated with log *OR*_2_ (metaregression beta coefficient=-0.325, p=0.016). We performed stratification analysis on wet AMD and the combined AMD of the *HTRA1* rs11200638 G→A polymorphism, and found a considerable difference in effects between wet AMD (Bayesian random effect *OR*_1_=10.110, 95% *CrI*: 6.998, 16.490; Bayesian random effect *OR*_2_=2.647, 95% *CrI*: 2.132, 3.280; λ=0.420, 95% *CrI*: 0.0.350, 0.491) and combined AMD (Bayesian random effect *OR*_1_=7.087, 95% *CrI*: 5.284, 9.523; Bayesian random effect *OR*_2_=1.931, 95% *CrI*: 1.643, 2.277; λ=0.337, 95% *CrI*: 0.267, 0.408). This stratification exhibited no between-study heterogeneity for either *OR*_1_ (*Q*=3.232, p=0.664, *I*^2^=0.0%) or *OR*_2_ (*Q*=0.890, p=0.971, *I*^2^=0.0%) for combined AMD, and found a moderate degree of between-study heterogeneity for *OR*_1_ (*Q*=13.978, p=0.030, *I*^2^=57.1%) and non-significant between-study heterogeneity for *OR*_2_ (*Q*=7.309, p=0.293, *I*^2^=17.9%) of the wet AMD (Table 5).

**Table 5:** Age related macular degeneration (AMD): *HTRA1* SNPs versus *ARMS2* single nucleotide polymorphisms (SNPs).

**Comparison**	**No. of studies**	**Total sample size (n)**	**Bayesian random effects**		**Fixed effects**		**Random effects**		**Heterogeneity**		
**Odds ratio**	**95% credible interval**	**Odds ratio**	**95% CI**	**Odds ratio**	**95% CI**	**Q**	**P value**	**I2 (%)**
***HTRA1* (rs11200638)**											
**A allele versus G allele**											
Total	16	4034/3212	/	/	2.664	2.476, 2.867	2.803	2.486, 3.159	34.576	0.003	56.6
HWE	14	3124/2784	/	/	2.754	2.542, 2.984	2.909	2.547, 3.324	30.471	0.004	57.4
Adjusted HWE	16	4034/3212	/	/	2.701	2.510, 2.907	2.827	2.519, 3.173	31.923	0.007	53
East Asian	7	835/868	/	/	2.847	2.473, 3.278	2.847	2.473, 3.278	4.873	0.56	0
Caucasian	8	2970/2160	/	/	2.589	2.366, 2.833	2.8	2.289, 3.424	28.452	0	75.4
Seven studies with 2 SNPs	7	1533/1206	/	/	3.059	2.717, 3.444	3.053	2.681, 3.478	7.028	0.318	14.7
**AA versus GG**											
Total	16	4034/3212	7.972	6.453, 9.778	7.21	6.035, 8.614	7.737	6.096, 9.821	24.308	0.06	39.2
HWE	14	3124/2784	8.424	6.667, 10.540	7.515	6.201, 9.107	8.16	6.314, 10.546	20.51	0.083	37.6
Adjusted HWE	16	4034/3212	8.225	6.700, 10.030	7.423	6.205, 8.880	7.928	6.286, 10.000	22.776	0.089	35.3
East Asian	7	835/868	7.604	5.541, 10.100	7.273	5.390, 9.815	7.273	5.390, 9.815	4.27	0.64	0
Caucasian	8	2970/2160	8.691	5.813, 13.600	7.258	5.719, 9.211	8.687	5.556, 13.582	19.969	0.006	65.8
Wet AMD	8	1348/1212	9.484	6.834, 12.800	9.205	6.941, 12.207	9.843	6.539, 14.817	14.147	0.049	51.6
Wet AMD + Dry AMD	8	2686/2000	6.561	5.137, 8.270	6.138	4.881, 7.719	6.138	4.881, 7.719	5.391	0.612	0
Seven studies investigated 2 SNPs	7	1533/1206	8.967	5.964, 12.920	8.534	6.411, 11.360	9.309	5.924, 14.682	14.097	0.029	58.8
**AG versus GG**											
Total	16	4034/3212	2.226	1.982, 2.496	2.168	1.944, 2.418	2.18	1.943, 2.447	15.784	0.397	5
HWE	14	3124/2784	2.28	2.006, 2.589	2.193	1.943, 2.475	2.232	1.935, 2.574	15.295	0.289	15.1
Adjusted HWE	16	4034/3212	2.252	2.011, 2.516	2.192	1.966, 2.445	2.204	1.962, 2.477	15.942	0.386	5.9
East Asian	7	835/868	2.277	1.781, 2.866	2.202	1.684, 2.878	2.219	1.665, 2.957	6.812	0.339	12.3
Caucasian	8	2970/2160	2.273	1.916, 2.741	2.205	1.948, 2.495	2.221	1.949, 2.531	7.453	0.383	6.1
Wet AMD	8	1348/1212	2.692	2.197, 3.249	2.706	2.231, 3.281	2.708	2.219, 3.305	7.311	0.397	4.3
Wet AMD + Dry AMD	8	2686/2000	1.959	1.723, 2.222	1.953	1.711, 2.230	1.953	1.711, 2.230	1.024	0.994	0
Seven studies with 2 SNPs	7	1533/1206	2.392	1.938, 2.907	2.396	2.004, 2.866	2.471	1.946, 3.138	9.387	0.153	36.2
**λ**											
Total			0.386	0.343, 0.430							
HWE			0.387	0.340, 0.435							
Adjusted HWE			0.386	0.343, 0.429							
East Asian			0.403	0.311, 0.491							
Caucasian			0.381	0.327, 0.438							
Wet AMD			0.441	0.373, 0.510							
Wet AMD + Dry AMD			0.359	0.300, 0.419							
Seven studies with 2 SNPs			0.4	0.331, 0.471							
***LOC387715/ARMS2* (rs10490924)**											
**T allele versus G allele**											
Total	18	5027/5878	/	/	2.725	2.556, 2.906	2.734	2.366, 3.158	80.195	0	78.8
HWE	17	4893/5744	/	/	2.742	2.569, 2.928	2.761	2.376, 3.209	79.116	0	79.8
Adjusted HWE	18	5027/5878	/	/	2.715	2.547, 2.896	2.719	2.351, 3.145	81.68	0	79.2
East Asian	3	289/325	/	/	2.692	2.086, 3.315	2.692	2.086, 3.315	0.481	0.786	0
Caucasian	13	4425/4355	/	/	2.769	2.580, 2.972	2.794	2.333, 3.346	73.265	0	83.6
Seven studies with 2 SNPs	7	1471/1225	/	/	3.276	2.912, 3.686	3.211	2.711, 3.802	11.596	0.072	48.3
**TT versus GG**											
Total	18	5027/5878	7.512	5.703, 9.659	7.096	6.069, 8.296	7.216	5.492, 9.480	48.208	0	65.3
HWE	17	4893/5744	7.826	5.886, 10.140	7.394	6.294, 8.683	7.533	5.707, 9.943	43.926	0	64.2
Adjusted HWE	18	5027/5878	7.51	5.692, 9.672	7.1	6.071, 8.303	7.209	5.483, 9.480	48.331	0	65.4
East Asian	3	289/325	/	/	6.934	4.206, 11.431	6.934	4.206, 11.431	1.232	0.54	0
Caucasian	13	4425/4355	7.57	5.326, 10.850	7.261	6.076, 8.677	7.41	5.176, 10.607	45.035	0	73.8
Wet AMD	7	1029/1073	8.567	5.509, 12.600	7.828	5.786, 10.582	8.273	5.191, 13.185	13.738	0.033	56.9
Wet AMD + Dry AMD	11	3998/4805	7.021	4.678, 9.950	6.846	5.703, 8.218	6.708	4.734, 9.505	33.919	0	71.1
Seven studies with 2 SNPs	7	1471/1225	9.767	6.169, 14.480	9.134	6.951, 12.002	9.521	5.922, 15.307	17.209	0.009	65.6
**GT versus GG**											
Total	18	5027/5878	2.353	2.072, 2.665	2.336	2.134, 2.558	2.324	1.993, 2.709	42.812	0.001	60.3
HWE	17	4893/5744	2.38	2.093, 2.702	2.343	2.138, 2.569	2.336	1.990, 2.741	42.638	0	62.5
Adjusted HWE	18	5027/5878	2.334	2.058, 2.643	2.316	2.115, 2.535	2.29	1.956, 2.681	45.09	0	62.3
East Asian	3	289/325	/	/	1.843	1.203, 2.823	2.119	0.893, 5.029	7.783	0.02	74.5
Caucasian	13	4425/4355	2.424	2.062, 2.865	2.422	2.198, 2.669	2.445	2.082, 2.871	29.79	0.003	59.7
Wet AMD	7	1029/1073	2.519	1.983, 3.147	2.531	2.053, 3.122	2.519	1.813, 3.501	13.05	0.042	54
Wet AMD + Dry AMD	11	3998/4805	2.285	1.921, 2.694	2.293	2.074, 2.536	2.253	1.886, 2.691	29.067	0.001	65.6
Seven studies with 2 SNPs	7	1471/1225	2.507	1.999, 3.088	2.564	2.137, 3.076	2.567	2.065, 3.191	7.834	0.251	23.4
**λ**											
Total			0.426	0.387, 0.467							
HWE			0.423	0.384, 0.463							
Adjusted HWE			0.422	0.383, 0.462							
East Asian			/	/							
Caucasian			0.438	0.395, 0.483							
Wet AMD			0.433	0.354, 0.513							
Wet AMD + Dry AMD			0.428	0.382, 0.475							
Seven studies with 2 SNPs			0.406	0.341, 0.472							

Summary odds ratios of *HTRA1* rs11200638 polymorphism and *LOC387715/ARMS2* rs10490924 polymorphism.

We also performed stratification analysis on the wet AMD and combined AMD of the *LOC387715/ARMS2* rs10490924 G→T polymorphism, and found a considerable difference in effect between wet AMD (Bayesian random effect *OR*_1_=8.567, 95% *CrI*: 5.509, 12.600; Bayesian random effect *OR*_2_=2.519, 95% *CrI*: 1.983, 3.147; λ=0.433, 95% *CrI*: 0.354, 0.513) and combined AMD (Bayesian random effect *OR*_1_=7.021, 95% *CrI*: 7.021; Bayesian random effect *OR*_2_=2.285, 95% *CrI*: 1.921, 2.694; λ=0.428, 95% *CrI*: 0.382, 0.475). This stratification found no between-study heterogeneity for either *OR*_1_ (*Q*=5.391, p=0.612, *I*^2^=0.0%) or *OR*_2_ (*Q*=1.024, p=0.994, *I*^2^=0.0%) for combined AMD, and found a moderate degree of between- study heterogeneity for *OR*_1_ (*Q*=14.147, p=0.049, *I*^2^=51.6%) and non-significant between-study heterogeneity for *OR*_2_ (*Q*=7.311, p=0.397, *I*^2^=4.3%) of the wet AMD (Table 5).

**Figure 4:**
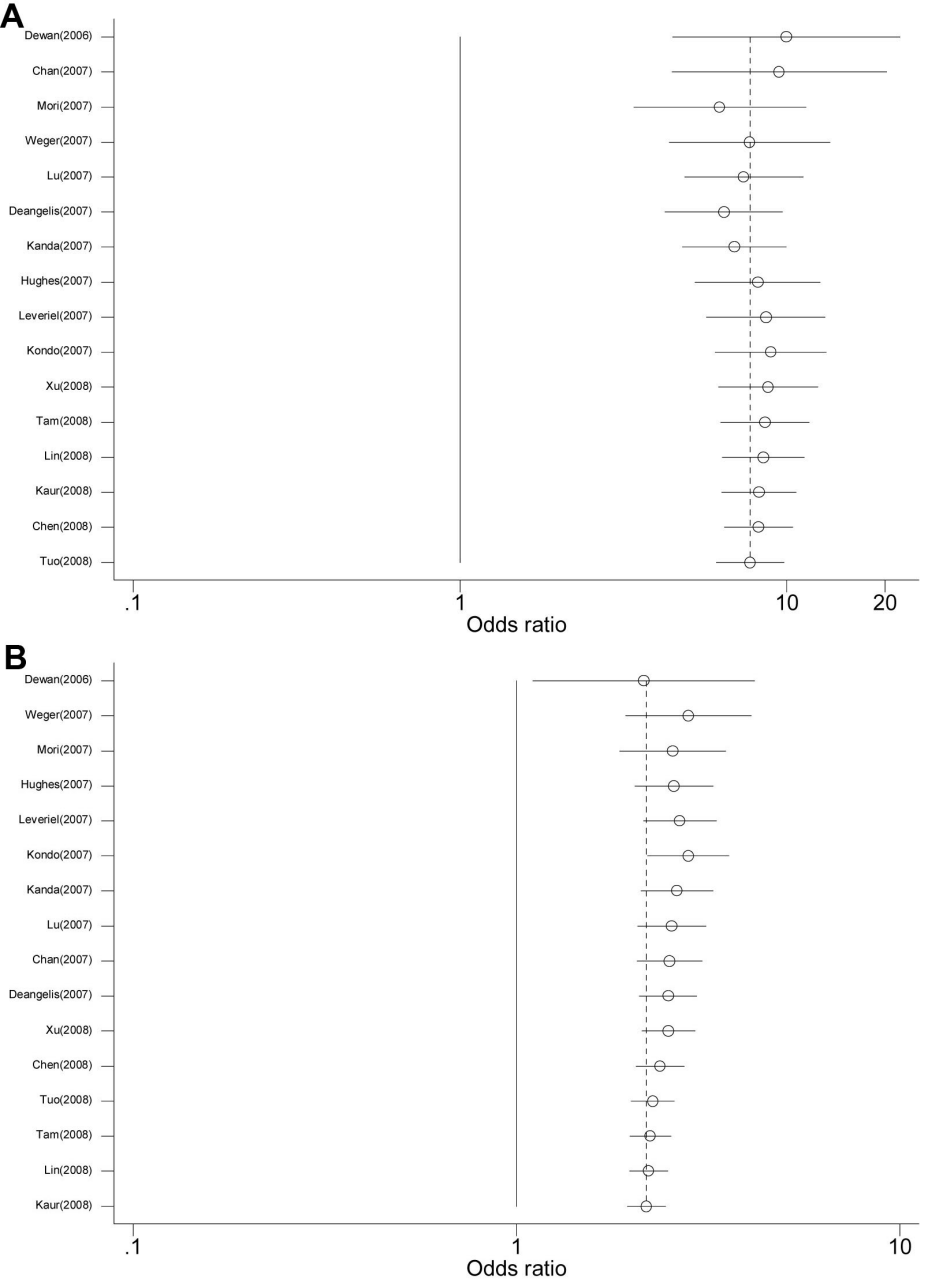
Cumulative random-effects meta-analysis of homozygous (**A**: AA versus GG) and heterozygous (**B**: AG versus GG) genotypes of the *HTRA1* gene rs11200638 G→A polymorphism and ager related macular degeneration (AMD). For study details, see Table 3.

**Figure 5:**
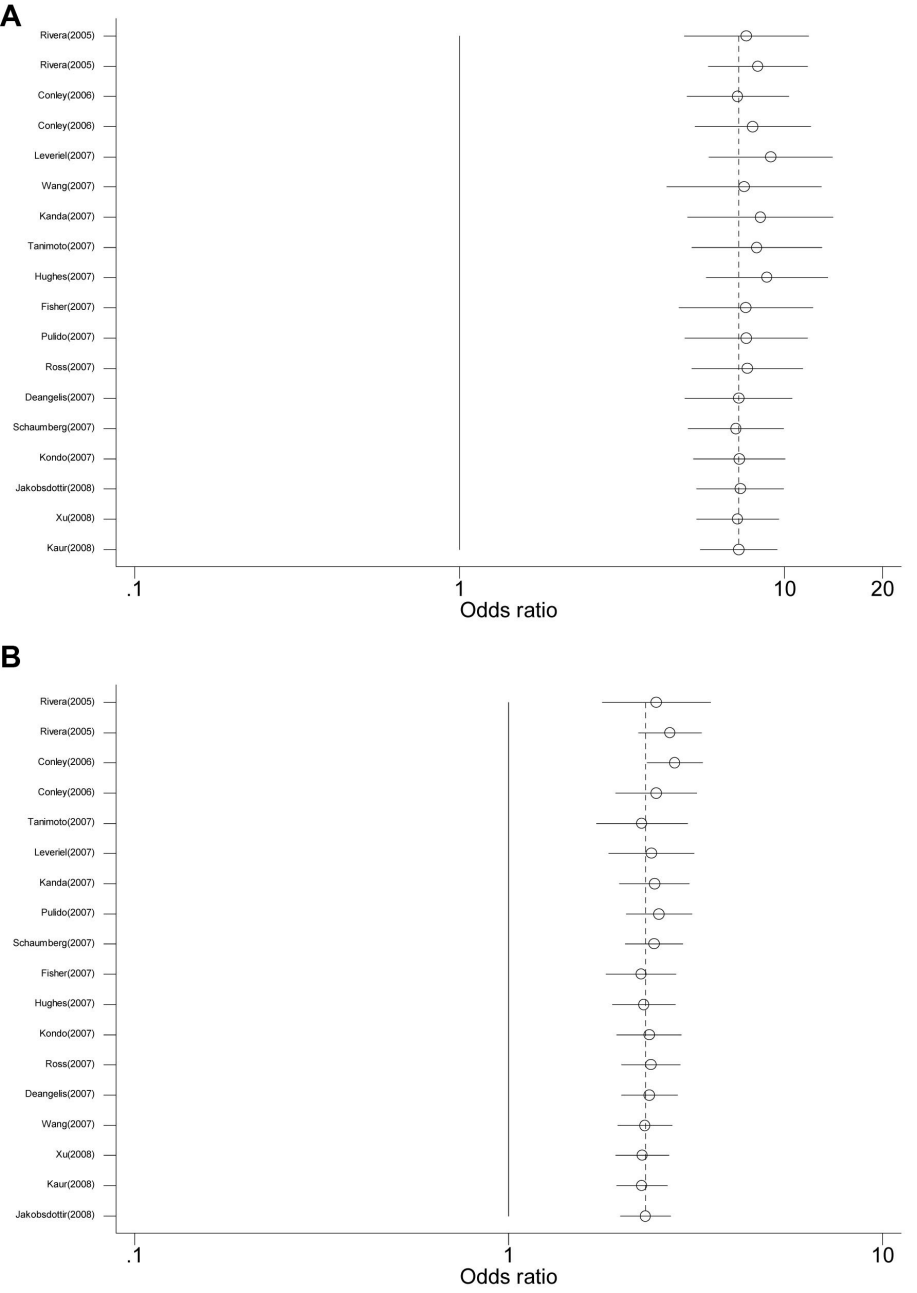
Cumulative random-effects meta-analysis of homozygous (**A**: TT versus GG) and heterozygous (**B**: GT versus GG) genotypes of *LOC387715/ARMS2* gene rs10490924 G→T polymorphism and age related macular degeneration (AMD). For study details, see Table 4.

There was no evidence of small study bias or publication bias for the two comparisons. For the *HTRA1* rs11200638 G→A polymorphism, funnel plots for the comparisons made for the AA homozygotes and AG heterozygotes gave corrected p=0.077 (corrected Begg’s *z*=1.77) and corrected p=0.669 (corrected Begg’s *z*=0.43), respectively. Figure 4 shows the cumulative result of meta-analysis of the AA homozygotes and AG heterozygotes; they remained significant and stayed relatively unchanged after the third study (Figure 4A,B). Figure 5 shows the cumulative result of meta-analysis of the TT homozygotes and TG heterozygotes of *LOC387715/ARMS2* rs10490924 with G→T polymorphism; they remained significant and relatively unchanged after the third study.

## Discussion

To our knowledge, this is the first general overview of the association between the *HTRA1* rs11200638 G→A polymorphism, the *LOC387715/ARMS2* rs10490924 G→T polymorphism, and susceptibility to AMD. The results of our meta-analysis suggest a strong association and a moderate codominant genetic mode of action. Our primary analysis shows that, for the *HTRA1* rs11200638 G→A polymorphism, the AA homozygotes carry an 8.5 fold increased risk of AMD, and the AG heterozygous variants carry just a 2.5 fold increase in risk when compared with GG homozygotes; for the *LOC387715/ARMS2* rs10490924 G→T polymorphism, the TT homozygotes carry a 7.5 fold increased risk of AMD, and the TG heterozygous variants carry just a 2.4 fold increase in risk when compared with the GG homozygotes. In addition, our allele-based analysis suggests a nearly 3.0-fold increase in susceptibility to AMD among persons with the A allele of the *HTRA1* rs11200638 G→A polymorphism and the T allele of the *LOC387715/ARMS2* rs10490924 G→T polymorphism.

Our findings were based on several gene-association studies, which include several thousand participants and were robust in terms of all the planned and performed sensitivity analyses. We found no evidence of publication bias or small study bias by funnel plots and cumulative meta-analysis; moreover, “moderate,” “moderate,” and “low” degrees of between-study heterogeneity were found in alleles (A versus G), homozygotes (AA versus GG), and heterozygotes (AG versus GG) of the association between the *HTRA1* rs11200638 G→A polymorphism and AMD. When HWE was examined, 11 of the 13 studies showed no deviation and two showed some deviation. The removal of the two HW disequilibrium studies meant that our overall results were also robust; statistical adjustment for the deviations were similar and consistent with the incipient results. The point estimate values were closer to a codominant model after removal of the HW disequilibrium studies and statistical adjustment for the deviation; this suggested a multiplicative genetic mode of action that needs to be verified by more studies, particularly large-scale, long-term longitudinal studies. Moderate between-study heterogeneity was also found in the alleles (T versus G), homozygotes (TT versus GG), and heterozygotes (TG versus GG) of the association between the *LOC387715/ARMS2* rs10490924 G→T polymorphism and AMD. However, the data we collected in this systematic review can only support a moderate codominant genetic model with a tight confidence interval.

The *HTRA1* gene encodes a member of the trypsin family of serine proteases [133]. The precise pathomechanism by which the HTRA1 rs11200638 A risk allele affects susceptibility to AMD is still unclear [134,135]. The upregulation of *HTRA1* plays a detrimental role in arthritic disease through its capacity to degrade extracellular matrices (ECMs) directly and to upregulate the expression of matrix metalloproteinase, which results in ECM degradation [88]. Yang et al. [90] hypothesized that the most likely mechanism in the involvement of rs11200638 with AMD may be the enhancement of ECM degradation [90]. As shown in the model of laser-induced CNV [136], the destruction of the Bruch membrane leads to CNV development [90]. Although the function of *HTRA1* in ocular tissues is unclear, it is reasonable to speculate that CNV may develop when the Bruch membrane is exposed to the detrimental effects of *HTRA1*. In vitro, higher luciferase expressions have been reported in both ARPE19 and HeLaS3 cells transfected with the *HTRA1* rs1120638 risk homozygote (AA) genotype when compared to the wild-type (GG) [101]. It has been suggested that the presence of the *HTRA1* rs11200638 A risk allele may alter the affinity of transcription factors, including the adaptor-related protein complex 2 alpha and serum response factor to the *HTRA1* promoter [101]. Another potential mechanism by which the *HTRA1* rs11200638 A allele may increase AMD risk is its ability to bind to TGF-β family members and to inhibit signaling of TGF-β family proteins, such as bone morphogenetic protein 2 and bone morphogenetic protein 4), which have previously been reported to act as negative growth regulators in RPE [89,137].

Although an association between the *HTRA1* rs11200638 G→A polymorphism and AMD was found, Kanda and others [95] considered that the rs11200638 G→A polymorphism of the *HTRA1* gene did not make a major contribution to regulation of the *HTRA1* gene and there is no association between *HTRA1* G→A polymorphism and AMD. To verify these conclusions, they generated mammalian expression constructs carrying three different lengths of the normal HTRA1 promoter (WT-long, -medium, and -short) and the mutant sequence carrying the AMD-risk allele at the single nucleotide polymorphism (SNP) rs11200638 (SNP-long and -medium), and these constructs were transfected into human embryonic kidney293 (HEK293), human-derived retinal pigment epithelial (ARPE19), and Human retinoblastoma (Y79) cells. As a result, they found that WT and variant SNPs of the *HTRA1* promoter activities did not show significant differences in the luciferase reporter expression, and the WT-short promoter (not including the rs11200638 region) showed higher transcriptional activities than the others. A further quantitative analysis provided no evidence for significant change of mRNA expression between control and AMD retinas. This finding contrasts with the previous original experiment, which suggested an increase in *HTRA1* expression in lymphocytes from AMD patients [90,127]. Taken together, these studies seem to draw a conflicting conclusion to those of the other studies in our meta-analysis.

Localization of the LOC387715/ARMS2 protein to the mitochondrial outer membrane in transfected mammalian cells suggests intriguing mechanisms through which an A69S change may influence AMD susceptibility. Mitochondria are implicated in the pathogenesis of other age-related neurodegenerative diseases, including Alzheimer disease, Parkinson disease, and so on [138]. Mitochondrial dysfunction associated with aging can result in impairment of the energy metabolism and homeostasis, generation of reactive oxygen species, accumulation of somatic mutations in mitochondrial DNA, and activation of the apoptotic pathway [139-141]. Decreased number and size of mitochondria, loss of cristae, or reduced matrix density are observed in AMD retinas compared with controls, and mitochondrial DNA deletions and cytochrome *c* oxidase-deficient cones accumulate in the aging retina, particularly in the macular region [140,142]. Moreover, mutations in mitochondrial proteins (e.g., dynamin-like guanosine triphosphatase [GTPase] optic atrophy 1 [OPA1]) are associated with optic neurodegenerative disorders [143]. Photoreceptors and RPE contain high levels of polyunsaturated fatty acids and are exposed to intense light and near-arterial levels of oxygen, providing considerable risk for oxidative damage [143,144]. Kanda and others therefore propose that the altered function of the putative mitochondrial protein LOC387715/ARMS2 by A69S substitution increases the susceptibility to the aging-associated generation of macular photoreceptors [95]. However, they did not observe any significant difference in the expression, stability, or localization of the A69S variant LOC387715/ARMS2 protein in mammalian cells. It is plausible that the A69S alteration modifies the function of the LOC387715/ARMS2 protein by affecting its conformation and/or interaction. For this reason, additional analysis of the LOC387715/ARMS2 protein with Ala or Ser codon 69 and its function in vivo are needed to better understand its contribution to AMD pathogenesis.

Even though the results presented here are contradictory, the A allele of the *HTRA1* gene rs11200638 G→A polymorphism is reasonably common, with an allele frequency of over 30% in a control population and over 40% in an Asian control population, and the T allele frequency of the *LOC387715/ARMS2* rs10490924 G→T polymorphism was 25.17% in a control population and 38.67% in Asians. This means that the effect at the population level, especially for Asian populations, could be quite important. The proportion of AA and AG genotypes of the *HTRA1* rs11200638 G→A polymorphism in a control population is 48% and the pooled OR for these two genotypes is 3.13. These two data were 64.07, 3.47 and 39.81, 3.07 for Asians and Caucasians, respectively. For the *LOC387715/ARMS2* rs10490924 G→T polymorphism, the proportion of TT and GT genotypes in a control population is 38.89% and the pooled *OR* for these two genotypes is 3.05. These two data were 64.00, 3.17 and 35.40, 3.13 for Asians and Caucasians, respectively.

The PAR for the combined genotypes AA and AG of the *HTRA1* rs11200638 G→A polymorphism is 56.0% (63.5% for Asians, 48.4% for Caucasians, 61.3% for wet AMD, 51.1% for combined AMD). The PAR for the combined genotypes TT and GT of *LOC387715/ARMS2* gene rs10490924 G→T polymorphism is 47.9% (55.4% for Asians, 44.1% for Caucasians, 56.8% for wet AMD, 42.4% for combined AMD). In other words, the *HTRA1* rs11200638 G→A polymorphism is involved in over half of all cases of AMD, quite close to the previous estimate of the first major AMD-susceptibility allele, *CFH* Y402H (58.9%) [79]. The *LOC387715/ARMS2* rs10490924 G→T polymorphism is also involved in nearly half of all AMD cases. Higher PAR can explain part of why both these genes (*HTRA1* rs11200638 G→A polymorphism and *LOC387715/ARMS2* rs10490924 G→T polymorphism) play important roles in AMD, especially for wet AMD populations.

In conclusion, this Human Genome Epidemiology (HuGE) systematic review presents strong evidence for an association between the *HTRA1* rs11200638 G→A polymorphism, *LOC387715/ARMS2* rs10490924 G→T polymorphism, and AMD, and suggests that both of these genes play important roles in this disease. Potential gene-gene and gene-environmental interactions and possible mechanisms of AMD are also summarized and discussed. Our findings suggest that these genetic variations may serve as biomarkers enabling the diagnosis of AMD in a more efficient and economical way. However, large-scale, long-term longitudinal studies are required to substantiate and strengthen this association.

## References

[1] Bandello F Lafuma A Berdeaux G Public health impact of neovascular age-related macular degeneration treatments extrapolated from visual acuity. Invest Ophthalmol Vis Sci 2007 48 96 103 17197522 10.1167/iovs.06-0283

[2] Bird AC The Bowman lecture. Towards an understanding of age-related macular disease. Eye 2003 17 457 66 12802343 10.1038/sj.eye.6700562

[3] Cruess AF Zlateva G Xu X Soubrane G Pauleikhoff D Lotery A Mones J Buggage R Schaefer C Knight T Goss TF Economic burden of bilateral neovascular age-related macular degeneration: multi-country observational study. Pharmacoeconomics 2008 26 57 73 18088159 10.2165/00019053-200826010-00006

[4] Evans JR Fletcher AE Wormald RP 28,000 Cases of age related macular degeneration causing visual loss in people aged 75 years and above in the United Kingdom may be attributable to smoking. Br J Ophthalmol 2005 89 550 3 15834082 10.1136/bjo.2004.049726 PMC1772624

[5] Klein R Klein BE Linton KL Prevalence of age-related maculopathy. The Beaver Dam Eye Study. Ophthalmology 1992 99 933 43 1630784 10.1016/s0161-6420(92)31871-8

[6] Muñoz B Klein R Rodriguez J Snyder R West SK Prevalence of age-related macular degeneration in a population-based sample of Hispanic people in Arizona: Proyecto VER. Arch Ophthalmol 2005 123 1575 80 16286621 10.1001/archopht.123.11.1575

[7] Owen CG Fletcher AE Donoghue M Rudnicka AR How big is the burden of visual loss caused by age related macular degeneration in the United Kingdom? Br J Ophthalmol 2003 87 312 7 12598445 10.1136/bjo.87.3.312 PMC1771556

[8] Rosenberg T Klie F The incidence of registered blindness caused by age-related macular degeneration. Acta Ophthalmol Scand 1996 74 399 402 8883559 10.1111/j.1600-0420.1996.tb00717.x

[9] Schick JH Iyengar SK Klein BE Klein R Reading K Liptak R Millard C Lee KE Tomany SC Moore EL Fijal BA Elston RC A whole-genome screen of a quantitative trait of age-related maculopathy in sibships from the Beaver Dam Eye Study. Am J Hum Genet 2003 72 1412 24 12717633 10.1086/375500 PMC1180302

[10] Soubrane G Coscas G Age-related macular degeneration. Rev Prat 1996 46 1722 9 8949279

[11] Vinding T Visual impairment of age-related macular degeneration. An epidemiological study of 1000 aged individuals. Acta Ophthalmol (Copenh) 1990 68 162 7 2356703 10.1111/j.1755-3768.1990.tb01898.x

[12] Vingerling JR Dielemans I Hofman A Grobbee DE Hijmering M Kramer CF de Jong PT The prevalence of age-related maculopathy in the Rotterdam Study. Ophthalmology 1995 102 205 10 7862408 10.1016/s0161-6420(95)31034-2

[13] Xu L Wang Y Li Y Wang Y Cui T Li J Jonas JB Causes of blindness and visual impairment in urban and rural areas in Beijing: the Beijing Eye Study. Ophthalmology 2006 113 1134.e1 11 16647133 10.1016/j.ophtha.2006.01.035

[14] Bonastre J Le Pen C Anderson P Ganz A Berto P Berdeaux G The epidemiology, economics and quality of life burden of age-related macular degeneration in France, Germany, Italy and the United Kingdom. Eur J Health Econ 2002 3 94 102 15609135 10.1007/s10198-002-0104-y

[15] Age-Related Eye Disease Study Research Group Risk factors associated with age-related macular degeneration. A case-control study in the age-related eye disease study: Age-Related Eye Disease Study Report Number 3. Ophthalmology 2000 107 2224 32 11097601 10.1016/s0161-6420(00)00409-7 PMC1470467

[16] Bressler NM Bressler SB Congdon NG Ferris FL 3rd Friedman DS Klein R Lindblad AS Milton RC Seddon JM Age-Related Eye Disease Study Research Group. Potential public health impact of Age-Related Eye Disease Study results: AREDS report no. 11. Arch Ophthalmol 2003 121 1621 4 14609922 10.1001/archopht.121.11.1621 PMC1473209

[17] Clemons TE Milton RC Klein R Seddon JM Ferris FL 3rd Age-Related Eye Disease Study Research Group. Risk factors for the incidence of Advanced Age-Related Macular Degeneration in the Age-Related Eye Disease Study (AREDS) AREDS report no. 19. Ophthalmology 2005 112 533 9 15808240 10.1016/j.ophtha.2004.10.047 PMC1513667

[18] Klein R Overview of progress in the epidemiology of age-related macular degeneration. Ophthalmic Epidemiol 2007 14 184 7 17896295 10.1080/09286580701344381

[19] Klein R Peto T Bird A Vannewkirk MR The epidemiology of age-related macular degeneration. Am J Ophthalmol 2004 137 486 95 15013873 10.1016/j.ajo.2003.11.069

[20] Seddon JM Chen CA The epidemiology of age-related macular degeneration. Int Ophthalmol Clin 2004 44 17 39 15577562 10.1097/00004397-200404440-00004

[21] Haddad S Chen CA Santangelo SL Seddon JM The genetics of age-related macular degeneration: a review of progress to date. Surv Ophthalmol 2006 51 316 63 16818082 10.1016/j.survophthal.2006.05.001

[22] Montezuma SR Sobrin L Seddon JM Review of genetics in age related macular degeneration. Semin Ophthalmol 2007 22 229 40 18097986 10.1080/08820530701745140

[23] Rakic JM Multifactorial influences on age-related macular degeneration. Bull Soc Belge Ophtalmol 2006 301 9 11 17552427

[24] Scholl HP Fleckenstein M Charbel Issa P Keilhauer C Holz FG Weber BH An update on the genetics of age-related macular degeneration. Mol Vis 2007 13 196 205 17327825 PMC2610372

[25] Tuo J Bojanowski CM Chan CC Genetic factors of age-related macular degeneration. Prog Retin Eye Res 2004 23 229 49 15094132 10.1016/j.preteyeres.2004.02.001 PMC1950336

[26] Asleh SA Chowers I Ethnic background as a risk factor for advanced age-related macular degeneration in Israel. Isr Med Assoc J 2007 9 656 8 17939627

[27] Francis PJ George S Schultz DW Rosner B Hamon S Ott J Weleber RG Klein ML Seddon JM The LOC387715 gene, smoking, body mass index, environmental associations with advanced age-related macular degeneration. Hum Hered 2007 63 212 8 17347568 10.1159/000100046

[28] Moeller SM Jacques PF Blumberg JB The potential role of dietary xanthophylls in cataract and age-related macular degeneration. J Am Coll Nutr 2000 19 522S 7S 11023002 10.1080/07315724.2000.10718975

[29] Seddon JM George S Rosner B Cigarette smoking, fish consumption, omega-3 fatty acid intake, and associations with age-related macular degeneration: the US Twin Study of Age-Related Macular Degeneration. Arch Ophthalmol 2006 124 995 1001 16832023 10.1001/archopht.124.7.995

[30] Seddon JM George S Rosner B Klein ML CFH gene variant, Y402H, and smoking, body mass index, environmental associations with advanced age-related macular degeneration. Hum Hered 2006 61 157 65 16816528 10.1159/000094141

[31] Arnarsson A Sverrisson T Stefánsson E Sigurdsson H Sasaki H Sasaki K Jonasson F Risk factors for five-year incident age-related macular degeneration: the Reykjavik Eye Study. Am J Ophthalmol 2006 142 419 28 16935586 10.1016/j.ajo.2006.04.015

[32] Chong EW Kreis AJ Wong TY Simpson JA Guymer RH Alcohol consumption and the risk of age-related macular degeneration: a systematic review and meta-analysis. Am J Ophthalmol 2008 145 707 15 18242575 10.1016/j.ajo.2007.12.005

[33] Douglas IJ Cook C Chakravarthy U Hubbard R Fletcher AE Smeeth L A case-control study of drug risk factors for age-related macular degeneration. Ophthalmology 2007 114 1164 9 17544775 10.1016/j.ophtha.2006.09.018

[34] Evans JR Risk factors for age-related macular degeneration. Prog Retin Eye Res 2001 20 227 53 11173253 10.1016/s1350-9462(00)00023-9

[35] Fraser-Bell S Wu J Klein R Azen SP Varma R Smoking, alcohol intake, estrogen use, and age-related macular degeneration in Latinos: the Los Angeles Latino Eye Study. Am J Ophthalmol 2006 141 79 87 16386980 10.1016/j.ajo.2005.08.024

[36] Knudtson MD Klein R Klein BE Alcohol consumption and the 15-year cumulative incidence of age-related macular degeneration. Am J Ophthalmol 2007 143 1026 9 17524768 10.1016/j.ajo.2007.01.036 PMC1950733

[37] Klaver CC Wolfs RC Assink JJ van Duijn CM Hofman A de Jong PT Genetic risk of age-related maculopathy. Population-based familial aggregation study. Arch Ophthalmol 1998 116 1646 51 9869796 10.1001/archopht.116.12.1646

[38] Luo L Harmon J Yang X Chen H Patel S Mineau G Yang Z Constantine R Buehler J Kaminoh Y Ma X Wong TY Zhang M Zhang K Familial aggregation of age-related macular degeneration in the Utah population. Vision Res 2008 48 494 500 18252239 10.1016/j.visres.2007.11.013

[39] Gottfredsdottir MS Sverrisson T Musch DC Stefánsson E Age related macular degeneration in monozygotic twins and their spouses in Iceland. Acta Ophthalmol Scand 1999 77 422 5 10463414 10.1034/j.1600-0420.1999.770413.x

[40] Grizzard SW Arnett D Haag SL Twin study of age-related macular degeneration. Ophthalmic Epidemiol 2003 10 315 22 14566632 10.1076/opep.10.5.315.17317

[41] Meyers SM A twin study on age-related macular degeneration. Trans Am Ophthalmol Soc 1994 92 775 843 7886884 PMC1298527

[42] Meyers SM Greene T Gutman FA A twin study of age-related macular degeneration. Am J Ophthalmol 1995 120 757 66 8540549 10.1016/s0002-9394(14)72729-1

[43] Seddon JM Cote J Page WF Aggen SH Neale MC The US twin study of age-related macular degeneration: relative roles of genetic and environmental influences. Arch Ophthalmol 2005 123 321 7 15767473 10.1001/archopht.123.3.321

[44] Allikmets R Shroyer NF Singh N Seddon JM Lewis RA Bernstein PS Peiffer A Zabriskie NA Li Y Hutchinson A Dean M Lupski JR Leppert M Mutation of the Stargardt disease gene (ABCR) in age-related macular degeneration. Science 1997 277 1805 7 9295268 10.1126/science.277.5333.1805

[45] De La Paz MA Guy VK Abou-Donia S Heinis R Bracken B Vance JM Gilbert JR Gass JD Haines JL Pericak-Vance MA Analysis of the Stargardt disease gene (ABCR) in age-related macular degeneration. Ophthalmology 1999 106 1531 6 10442900 10.1016/S0161-6420(99)90449-9

[46] Rivera A White K Stöhr H Steiner K Hemmrich N Grimm T Jurklies B Lorenz B Scholl HP Apfelstedt-Sylla E Weber BH A comprehensive survey of sequence variation in the ABCA4 (ABCR) gene in Stargardt disease and age-related macular degeneration. Am J Hum Genet 2000 67 800 13 10958763 10.1086/303090 PMC1287885

[47] Baird PN Richardson AJ Robman LD Dimitrov PN Tikellis G McCarty CA Guymer RH Apolipoprotein (APOE) gene is associated with progression of age-related macular degeneration (AMD). Hum Mutat 2006 27 337 42 16453339 10.1002/humu.20288

[48] Friedman DA Lukiw WJ Hill JM Apolipoprotein E epsilon4 offers protection against age-related macular degeneration. Med Hypotheses 2007 68 1047 55 17141963 10.1016/j.mehy.2006.09.049 PMC1857420

[49] Nowak M Swietochowska E Szapska B Marek B Wielkoszyński T Kozioł H Klimek J Kajdaniuk D Kos-Kudła B Ostrowska Z Karpe J Głogowska-Szelag J Siemińska L The apolipoprotein E polymorphism in age related macular degeneration. Klin Oczna 2004 106 427 8 15636224

[50] Schmidt S Saunders AM De La Paz MA Postel EA Heinis RM Agarwal A Scott WK Gilbert JR McDowell JG Bazyk A Gass JD Haines JL Pericak-Vance MA Association of the apolipoprotein E gene with age-related macular degeneration: possible effect modification by family history, age, and gender. Mol Vis 2000 6 287 93 11141572

[51] Simonelli F Margaglione M Testa F Cappucci G Manitto MP Brancato R Rinaldi E Apolipoprotein E polymorphisms in age-related macular degeneration in an Italian population. Ophthalmic Res 2001 33 325 8 11721184 10.1159/000055688

[52] Thakkinstian A Bowe S McEvoy M Smith W Attia J Association between apolipoprotein E polymorphisms and age-related macular degeneration: A HuGE review and meta-analysis. Am J Epidemiol 2006 164 813 22 16916985 10.1093/aje/kwj279

[53] Tuo J Ning B Bojanowski CM Lin ZN Ross RJ Reed GF Shen D Jiao X Zhou M Chew EY Kadlubar FF Chan CC Synergic effect of polymorphisms in ERCC6 5′ flanking region and complement factor H on age-related macular degeneration predisposition. Proc Natl Acad Sci USA 2006 103 9256 61 16754848 10.1073/pnas.0603485103 PMC1474016

[54] Stone EM Braun TA Russell SR Kuehn MH Lotery AJ Moore PA Eastman CG Casavant TL Sheffield VC Missense variations in the fibulin 5 gene and age-related macular degeneration. N Engl J Med 2004 351 346 53 15269314 10.1056/NEJMoa040833

[55] Fisher SA Rivera A Fritsche LG Keilhauer CN Lichtner P Meitinger T Rudolph G Weber BH Case-control genetic association study of fibulin-6 (FBLN6 or HMCN1) variants in age-related macular degeneration (AMD). Hum Mutat 2007 28 406 13 17216616 10.1002/humu.20464

[56] Schultz DW Weleber RG Lawrence G Barral S Majewski J Acott TS Klein ML HEMICENTIN-1 (FIBULIN-6) and the 1q31 AMD locus in the context of complex disease: review and perspective. Ophthalmic Genet 2005 26 101 5 16020313 10.1080/13816810590968023

[57] Ayyagari R Zhang K Hutchinson A Yu Z Swaroop A Kakuk LE Seddon JM Bernstein PS Lewis RA Tammur J Yang Z Li Y Zhang H Yashar BM Liu J Petrukhin K Sieving PA Allikmets R Evaluation of the ELOVL4 gene in patients with age-related macular degeneration. Ophthalmic Genet 2001 22 233 9 11803489 10.1076/opge.22.4.233.2219

[58] DeAngelis MM Ji F Kim IK Adams S Capone A Jr Ott J Miller JW Dryja TP Cigarette smoking, CFH, APOE, ELOVL4, and risk of neovascular age-related macular degeneration. Arch Ophthalmol 2007 125 49 54 17210851 10.1001/archopht.125.1.49

[59] Seitsonen S Lemmelä S Holopainen J Tommila P Ranta P Kotamies A Moilanen J Palosaari T Kaarniranta K Meri S Immonen I Järvelä I Analysis of variants in the complement factor H, the elongation of very long chain fatty acids-like 4 and the hemicentin 1 genes of age-related macular degeneration in the Finnish population. Mol Vis 2006 12 796 801 16885922

[60] Gold B Merriam JE Zernant J Hancox LS Taiber AJ Gehrs K Cramer K Neel J Bergeron J Barile GR Smith RT AMD Genetics Clinical Study Group, Hageman GS, Dean M, Allikmets R. Variation in factor B (BF) and complement component 2 (C2) genes is associated with age-related macular degeneration. Nat Genet 2006 38 458 62 16518403 10.1038/ng1750 PMC2921703

[61] Despriet DD Bergen AA Merriam JE Zernant J Barile GR Smith RT Barbazetto IA van Soest S Bakker A de Jong PT Allikmets R Klaver CC Comprehensive analysis of the candidate genes CCL2, CCR2, and TLR4 in age-related macular degeneration. Invest Ophthalmol Vis Sci 2008 49 364 71 18172114 10.1167/iovs.07-0656 PMC2754756

[62] Kaur I Hussain A Hussain N Das T Pathangay A Mathai A Hussain A Nutheti R Nirmalan PK Chakrabarti S Analysis of CFH, TLR4, and APOE polymorphism in India suggests the Tyr402His variant of CFH to be a global marker for age-related macular degeneration. Invest Ophthalmol Vis Sci 2006 47 3729 35 16936080 10.1167/iovs.05-1430

[63] Zareparsi S Buraczynska M Branham KE Shah S Eng D Li M Pawar H Yashar BM Moroi SE Lichter PR Petty HR Richards JE Abecasis GR Elner VM Swaroop A Toll-like receptor 4 variant D299G is associated with susceptibility to age-related macular degeneration. Hum Mol Genet 2005 14 1449 55 15829498 10.1093/hmg/ddi154

[64] Churchill AJ Carter JG Lovell HC Ramsden C Turner SJ Yeung A Escardo J Atan D VEGF polymorphisms are associated with neovascular age-related macular degeneration. Hum Mol Genet 2006 15 2955 61 16940309 10.1093/hmg/ddl238

[65] Fisher SA Abecasis GR Yashar BM Zareparsi S Swaroop A Iyengar SK Klein BE Klein R Lee KE Majewski J Schultz DW Klein ML Seddon JM Santangelo SL Weeks DE Conley YP Mah TS Schmidt S Haines JL Pericak-Vance MA Gorin MB Schulz HL Pardi F Lewis CM Weber BH Meta-analysis of genome scans of age-related macular degeneration. Hum Mol Genet 2005 14 2257 64 15987700 10.1093/hmg/ddi230

[66] Barral S Francis PJ Schultz DW Schain MB Haynes C Majewski J Ott J Acott T Weleber RG Klein ML Expanded genome scan in extended families with age-related macular degeneration. Invest Ophthalmol Vis Sci 2006 47 5453 9 17122136 10.1167/iovs.06-0655

[67] Santangelo SL Yen CH Haddad S Fagerness J Huang C Seddon JM A discordant sib-pair linkage analysis of age-related macular degeneration. Ophthalmic Genet 2005 26 61 7 16020308 10.1080/13816810490967944

[68] Seddon JM Santangelo SL Book K Chong S Cote J A genomewide scan for age-related macular degeneration provides evidence for linkage to several chromosomal regions. Am J Hum Genet 2003 73 780 90 12945014 10.1086/378505 PMC1180601

[69] Kim NR Kang JH Kwon OW Lee SJ Oh JH Chin HS Association between complement factor H gene polymorphisms and neovascular age-related macular degeneration in Koreans. Invest Ophthalmol Vis Sci 2008 49 2071 6 18223247 10.1167/iovs.07-1195

[70] Francis PJ Schultz DW Hamon S Ott J Weleber RG Klein ML Haplotypes in the Complement Factor H (CFH) Gene: Associations with Drusen and Advanced Age-Related Macular Degeneration. PLoS ONE 2007 2 e1197 18043728 10.1371/journal.pone.0001197 PMC2077927

[71] Kaare M Seitsonen S Jarvela I Meri S Laivuori H Complement factor H Y402H polymorphism and characteristics of exudative age-related macular degeneration lesions. Acta Ophthalmol Scand 2008 86 390 4 10.1111/j.1600-0420.2007.01050.x 17995985

[72] Wegscheider BJ Weger M Renner W Steinbrugger I März W Mossböck G Temmel W El-Shabrawi Y Schmut O Jahrbacher R Haas A Association of complement factor H Y402H gene polymorphism with different subtypes of exudative age-related macular degeneration. Ophthalmology 2007 114 738 42 17398321 10.1016/j.ophtha.2006.07.048

[73] Narayanan R Butani V Boyer DS Atilano SR Resende GP Kim DS Chakrabarti S Kuppermann BD Khatibi N Chwa M Nesburn AB Kenney MC Complement factor H polymorphism in age-related macular degeneration. Ophthalmology 2007 114 1327 31 17306880 10.1016/j.ophtha.2006.10.035

[74] Chen LJ Liu DT Tam PO Chan WM Liu K Chong KK Lam DS Pang CP Association of complement factor H polymorphisms with exudative age-related macular degeneration. Mol Vis 2006 12 1536 42 17167412

[75] Baird PN Islam FM Richardson AJ Cain M Hunt N Guymer R Analysis of the Y402H variant of the complement factor H gene in age-related macular degeneration. Invest Ophthalmol Vis Sci 2006 47 4194 8 17003406 10.1167/iovs.05-1285

[76] Maller J George S Purcell S Fagerness J Altshuler D Daly MJ Seddon JM Common variation in three genes, including a noncoding variant in CFH, strongly influences risk of age-related macular degeneration. Nat Genet 2006 38 1055 9 16936732 10.1038/ng1873

[77] Souied EH Leveziel N Richard F Dragon-Durey MA Coscas G Soubrane G Benlian P Fremeaux-Bacchi V Y402H complement factor H polymorphism associated with exudative age-related macular degeneration in the French population. Mol Vis 2005 11 1135 40 16379025

[78] Klein RJ Zeiss C Chew EY Tsai JY Sackler RS Haynes C Henning AK SanGiovanni JP Mane SM Mayne ST Bracken MB Ferris FL Ott J Barnstable C Hoh J Complement factor H polymorphism in age-related macular degeneration. Science 2005 308 385 9 15761122 10.1126/science.1109557 PMC1512523

[79] Thakkinstian A Han P McEvoy M Smith W Hoh J Magnusson K Zhang K Attia J Systematic review and meta-analysis of the association between complement factor H Y402H polymorphisms and age-related macular degeneration. Hum Mol Genet 2006 15 2784 90 16905558 10.1093/hmg/ddl220

[80] Okamoto H Umeda S Obazawa M Minami M Noda T Mizota A Honda M Tanaka M Koyama R Takagi I Sakamoto Y Saito Y Miyake Y Iwata T Complement factor H polymorphisms in Japanese population with age-related macular degeneration. Mol Vis 2006 12 156 8 16541016

[81] Uka J Tamura H Kobayashi T Yamane K Kawakami H Minamoto A Mishima HK No association of complement factor H gene polymorphism and age-related macular degeneration in the Japanese population. Retina 2006 26 985 7 17151483 10.1097/01.iae.0000244068.18520.3e

[82] Schmidt S Hauser MA Scott WK Postel EA Agarwal A Gallins P Wong F Chen YS Spencer K Schnetz-Boutaud N Haines JL Pericak-Vance MA Cigarette smoking strongly modifies the association of LOC387715 and age-related macular degeneration. Am J Hum Genet 2006 78 852 64 16642439 10.1086/503822 PMC1474047

[83] Jakobsdottir J Conley YP Weeks DE Mah TS Ferrell RE Gorin MB Susceptibility genes for age-related maculopathy on chromosome 10q26. Am J Hum Genet 2005 77 389 407 16080115 10.1086/444437 PMC1226205

[84] Rivera A Fisher SA Fritsche LG Keilhauer CN Lichtner P Meitinger T Weber BH Hypothetical LOC387715 is a second major susceptibility gene for age-related macular degeneration, contributing independently of complement factor H to disease risk. Hum Mol Genet 2005 14 3227 36 16174643 10.1093/hmg/ddi353

[85] Tocharus J Tsuchiya A Kajikawa M Ueta Y Oka C Kawaichi M Developmentally regulated expression of mouse HtrA3 and its role as an inhibitor of TGF-beta signaling. Dev Growth Differ 2004 46 257 74 15206957 10.1111/j.1440-169X.2004.00743.x

[86] Miyake K Horikawa Y Hara K Yasuda K Osawa H Furuta H Hirota Y Yamagata K Hinokio Y Oka Y Iwasaki N Iwamoto Y Yamada Y Seino Y Maegawa H Kashiwagi A Yamamoto K Tokunaga K Takeda J Makino H Nanjo K Kadowaki T Kasuga M Association of TCF7L2 polymorphisms with susceptibility to type 2 diabetes in 4,087 Japanese subjects. J Hum Genet 2008 53 174 80 18097733 10.1007/s10038-007-0231-5

[87] Ly DH Lockhart DJ Lerner RA Schultz PG Mitotic misregulation and human aging. Science 2000 287 2486 92 10741968 10.1126/science.287.5462.2486

[88] Grau S Richards PJ Kerr B Hughes C Caterson B Williams AS Junker U Jones SA Clausen T Ehrmann M The role of human HtrA1 in arthritic disease. J Biol Chem 2006 281 6124 9 16377621 10.1074/jbc.M500361200

[89] Oka C Tsujimoto R Kajikawa M Koshiba-Takeuchi K Ina J Yano M Tsuchiya A Ueta Y Soma A Kanda H Matsumoto M Kawaichi M HtrA1 serine protease inhibits signaling mediated by Tgfbeta family proteins. Development 2004 131 1041 53 14973287 10.1242/dev.00999

[90] Yang Z Camp NJ Sun H Tong Z Gibbs D Cameron DJ Chen H Zhao Y Pearson E Li X Chien J Dewan A Harmon J Bernstein PS Shridhar V Zabriskie NA Hoh J Howes K Zhang K A variant of the HTRA1 gene increases susceptibility to age-related macular degeneration. Science 2006 314 992 3 17053109 10.1126/science.1133811

[91] Cameron DJ Yang Z Gibbs D Chen H Kaminoh Y Jorgensen A Zeng J Luo L Brinton E Brinton G Brand JM Bernstein PS Zabriskie NA Tang S Constantine R Tong Z Zhang K HTRA1 variant confers similar risks to geographic atrophy and neovascular age-related macular degeneration. Cell Cycle 2007 6 1122 5 17426452 10.4161/cc.6.9.4157

[92] Cameron DJ Yang Z Tong Z Zhao Y Praggastis A Brinton E Harmon J Chen Y Pearson E Bernstein PS Brinton G Li X Jorgensen A Schneider S Gibbs D Chen H Wang C Howes K Camp NJ Zhang K 10q26 is associated with increased risk of age-related macular degeneration in the Utah population. Adv Exp Med Biol 2008 613 253 8 18188952 10.1007/978-0-387-74904-4_29

[93] Pulido JS Peterson LM Mutapcic L Bryant S Highsmith WE LOC387715/HTRA1 and complement factor H variants in patients with age-related macular degeneration seen at the mayo clinic. Ophthalmic Genet 2007 28 203 7 18161619 10.1080/13816810701649617

[94] Gibbs D Yang Z Constantine R Ma X Camp NJ Yang X Chen H Jorgenson A Hau V Dewan A Zeng J Harmon J Buehler J Brand JM Hoh J Cameron DJ Dixit M Tong Z Zhang K Further mapping of 10q26 supports strong association of HTRA1 polymorphisms with age-related macular degeneration. Vision Res 2008 48 685 9 18207215 10.1016/j.visres.2007.10.022

[95] Kanda A Chen W Othman M Branham KE Brooks M Khanna R He S Lyons R Abecasis GR Swaroop A A variant of mitochondrial protein LOC387715/ARMS2, not HTRA1, is strongly associated with age-related macular degeneration. Proc Natl Acad Sci USA 2007 104 16227 32 17884985 10.1073/pnas.0703933104 PMC1987388

[96] Deangelis MM Ji F Adams S Morrison MA Harring AJ Sweeney MO Capone A Jr Miller JW Dryja TP Ott J Kim IK Alleles in the HtrA Serine Peptidase 1 Gene Alter the Risk of Neovascular Age-Related Macular Degeneration. Ophthalmology 2008 115 1209 15 18164066 10.1016/j.ophtha.2007.10.032 PMC4242506

[97] Chen H Yang Z Gibbs D Yang X Hau V Zhao P Ma X Zeng J Luo L Pearson E Constantine R Kaminoh Y Harmon J Tong Z Stratton CA Cameron DJ Tang S Zhang K Association of HTRA1 polymorphism and bilaterality in advanced age-related macular degeneration. Vision Res 2008 48 690 4 18206206 10.1016/j.visres.2007.10.014

[98] Weger M Renner W Steinbrugger I Köfer K Wedrich A Groselj-Strele A El-Shabrawi Y Schmut O Haas A Association of the HTRA1 −625G>A promoter gene polymorphism with exudative age-related macular degeneration in a Central European population. Mol Vis 2007 13 1274 9 17679948

[99] Leveziel N Souied EH Richard F Barbu V Zourdani A Morineau G Zerbib J Coscas G Soubrane G Benlian P PLEKHA1–LOC387715-HTRA1 polymorphisms and exudative age-related macular degeneration in the French population. Mol Vis 2007 13 2153 9 18079691

[100] Hughes AE Orr N Patterson C Esfandiary H Hogg R McConnell V Silvestri G Chakravarthy U Neovascular age-related macular degeneration risk based on CFH, LOC387715/HTRA1, and smoking. PLoS Med 2007 4 e355 18162041 10.1371/journal.pmed.0040355 PMC2222948

[101] Dewan A Liu M Hartman S Zhang SS Liu DT Zhao C Tam PO Chan WM Lam DS Snyder M Barnstable C Pang CP Hoh J HTRA1 promoter polymorphism in wet age-related macular degeneration. Science 2006 314 989 92 17053108 10.1126/science.1133807

[102] Lu F Hu J Zhao P Lin Y Yang Y Liu X Fan Y Chen B Liao S Du Q Lei C Cameron DJ Zhang K Yang Z HTRA1 variant increases risk to neovascular age-related macular degeneration in Chinese population. Vision Res 2007 47 3120 3 17904186 10.1016/j.visres.2007.08.010

[103] Lin JM Wan L Tsai YY Lin HJ Tsai Y Lee CC Tsai CH Tsai FJ Tseng SH HTRA1 polymorphism in dry and wet age-related macular degeneration. Retina 2008 28 309 13 18301036 10.1097/IAE.0b013e31814cef3a

[104] Tam PO Ng TK Liu DT Chan WM Chiang SW Chen LJ DeWan A Hoh J Lam DS Pang CP HTRA1 Variants in Exudative Age-related Macular Degeneration and Interactions with Smoking and CFH. Invest Ophthalmol Vis Sci 2008 49 2357 65 18316707 10.1167/iovs.07-1520 PMC3215269

[105] Mori K Horie-Inoue K Kohda M Kawasaki I Gehlbach PL Awata T Yoneya S Okazaki Y Inoue S Association of the HTRA1 gene variant with age-related macular degeneration in the Japanese population. J Hum Genet 2007 52 636 41 17568988 10.1007/s10038-007-0162-1

[106] Kondo N Honda S Ishibashi K Tsukahara Y Negi A LOC387715/HTRA1 variants in polypoidal choroidal vasculopathy and age-related macular degeneration in a Japanese population. Am J Ophthalmol 2007 144 608 12 17692272 10.1016/j.ajo.2007.06.003

[107] Yoshida T DeWan A Zhang H Sakamoto R Okamoto H Minami M Obazawa M Mizota A Tanaka M Saito Y Takagi I Hoh J Iwata T HTRA1 promoter polymorphism predisposes Japanese to age-related macular degeneration. Mol Vis 2007 13 545 8 17438519 PMC2652018

[108] Kaur I Katta S Hussain A Hussain N Mathai A Narayanan R Hussain A Reddy RK Majji AB Das T Chakrabarti S Variants in the 10q26 Gene Cluster (LOC387715 and HTRA1) Exhibit Enhanced Risk of Age-Related Macular Degeneration along with CFH in Indian Patients. Invest Ophthalmol Vis Sci 2008 49 1771 6 18436811 10.1167/iovs.07-0560

[109] Rivera A Fisher SA Fritsche LG Keilhauer CN Lichtner P Meitinger T Weber BH Hypothetical LOC387715 is a second major susceptibility gene for age-related macular degeneration, contributing independently of complement factor H to disease risk. Hum Mol Genet 2005 14 3227 36 16174643 10.1093/hmg/ddi353

[110] Egger M Juni P Bartlett C Holenstein F Sterne J How important are comprehensive literature searches and the assessment of trial quality in systematic reviews? Empirical study. Health Technol Assess 2003 7 1 76 12583822

[111] Stroup DF Berlin JA Morton SC Olkin I Williamson GD Rennie D Moher D Becker BJ Sipe TA Thacker SB Meta-analysis of observational studies in epidemiology: a proposal for reporting. Meta-analysis Of Observational Studies in Epidemiology (MOOSE) group. JAMA 2000 283 2008 12 10789670 10.1001/jama.283.15.2008

[112] Chang YC Chang TJ Jiang YD Kuo SS Lee KC Chiu KC Chuang LM Association study of the genetic polymorphisms of the transcription factor 7-like 2 (TCF7L2) gene and type 2 diabetes in the Chinese population. Diabetes 2007 56 2631 7 17579206 10.2337/db07-0421

[113] Mantel N Haenszel W Statistical aspects of the analysis of data from retrospective studies of disease. J Natl Cancer Inst 1959 22 719 48 13655060

[114] DerSimonian R Laird N Meta-analysis in clinical trials. Control Clin Trials 1986 7 177 88 3802833 10.1016/0197-2456(86)90046-2

[115] Lau J Ioannidis JP Schmid CH Quantitative synthesis in systematic reviews. Ann Intern Med 1997 127 820 6 9382404 10.7326/0003-4819-127-9-199711010-00008

[116] Minelli C Thompson JR Abrams KR Lambert PC Bayesian implementation of a genetic model-free approach to the meta-analysis of genetic association studies. Stat Med 2005 24 3845 61 16320276 10.1002/sim.2393

[117] Emigh T A comparison of tests for Hardy-Weinberg equilibrium. Biometrics 1980 36 627 42 25856832

[118] Trikalinos TA Salanti G Khoury MJ Ioannidis JP Impact of violations and deviations in Hardy-Weinberg equilibrium on postulated gene-disease associations. Am J Epidemiol 2006 163 300 9 16410351 10.1093/aje/kwj046

[119] Sterne J, Bradburn M, Egger M. Meta-analysis in Stata. In: Egger M, Davey Smith G, Altman D, editors. Systematic reviews in health care. 2nd ed. Boston, MA: Blackwell BMJ Books; 2001. p. 347–69.

[120] Higgins JP Thompson SG Deeks JJ Altman DG Measuring inconsistency in meta-analyses. BMJ 2003 327 557 60 12958120 10.1136/bmj.327.7414.557 PMC192859

[121] Higgins JP Thompson SG Quantifying heterogeneity in a meta-analysis. Stat Med 2002 21 1539 58 12111919 10.1002/sim.1186

[122] Egger M Davey Smith G Schneider M Minder C Bias in meta-analysis detected by a simple, graphical test. BMJ 1997 315 629 34 9310563 10.1136/bmj.315.7109.629 PMC2127453

[123] Lau J Antman EM Jimenez-Silva J Kupelnick B Mosteller F Chalmers TC Cumulative meta-analysis of therapeutic trials for myocardial infarction. N Engl J Med 1992 327 248 54 1614465 10.1056/NEJM199207233270406

[124] Stata Corporation. Stata statistical software, release 9.0. College Station, TX: Stata Corporation 2005.

[125] Spiegelhalter D, Thomas A, Best N, Lunn D. WinBUGS user manual. Version 1.4, January 2003. Cambridge, United Kingdom: MRC Biostatistics Unit, Institute of Public Health. 2003. http://www.mrc-bsu.cam.ac.uk/bugs/winbugs/manual14.pdf

[126] Canfield AE Hadfield KD Rock CF Wylie EC Wilkinson FL HtrA1: a novel regulator of physiological and pathological matrix mineralization? Biochem Soc Trans 2007 35 669 71 17635117 10.1042/BST0350669

[127] DeWan A Bracken MB Hoh J Two genetic pathways for age-related macular degeneration. Curr Opin Genet Dev 2007 17 228 33 17467263 10.1016/j.gde.2007.04.004

[128] Ross RJ Verma V Rosenberg KI Chan CC Tuo J Genetic markers and biomarkers for age-related macular degeneration. Expert Rev Ophthalmol 2007 2 443 57 17917691 10.1586/17469899.2.3.443 PMC2000850

[129] Marx J Gene offers insight into macular degeneration. Science 2006 314 405 17053121 10.1126/science.314.5798.405a

[130] Chan CC Shen D Zhou M Ross RJ Ding X Zhang K Green WR Tuo J Human HtrA1 in the archived eyes with age-related macular degeneration. Trans Am Ophthalmol Soc 2007105:92–7 18427598 PMC2258134

[131] Leveziel N Zerbib J Richard F Querques G Morineau G Fremeaux-Bacchi V Coscas G Soubrane G Benlian P Souied EH Genotype-phenotype correlations for exudative Age-related Macular Degeneration associated with homozygous HTRA1 and CFH genotypes. Invest Ophthalmol Vis Sci 2008 49 3090 4 18362109 10.1167/iovs.07-1540

[132] Montes T Goicoechea de Jorge E Ramos R Gomà M Pujol O Sánchez-Corral P Rodríguez de Córdoba S Genetic deficiency of complement factor H in a patient with age-related macular degeneration and membranoproliferative glomerulonephritis. Mol Immunol 2008 45 2897 904 18336910 10.1016/j.molimm.2008.01.027

[133] Pallen MJ Wren BW The HtrA family of serine proteases. Mol Microbiol 1997 26 209 21 9383148 10.1046/j.1365-2958.1997.5601928.x

[134] Thornton J Edwards R Mitchell P Harrison RA Buchan I Kelly SP Smoking and age-related macular degeneration: a review of association. Eye 2005 19 935 44 16151432 10.1038/sj.eye.6701978

[135] Conley YP Jakobsdottir J Mah T Weeks DE Klein R Kuller L Ferrell RE Gorin MB CFH, ELOVL4, PLEKHA1 and LOC387715 genes and susceptibility to age-related maculopathy: AREDS and CHS cohorts and meta-analyses. Hum Mol Genet 2006 15 3206 18 17000705 10.1093/hmg/ddl396

[136] Odergren A Ming Y Kvanta A Photodynamic therapy of experimental choroidal neovascularization in the mouse. Curr Eye Res 2006 31 765 74 16966149 10.1080/02713680600865045

[137] Mathura JR Jr Jafari N Chang JT Hackett SF Wahlin KJ Della NG Okamoto N Zack DJ Campochiaro PA Bone morphogenetic proteins-2 and −4: negative growth regulators in adult retinal pigmented epithelium. Invest Ophthalmol Vis Sci 2000 41 592 600 10670493

[138] Lin MT Beal MF Mitochondrial dysfunction and oxidative stress in neurodegenerative diseases. Nature 2006 443 787 95 17051205 10.1038/nature05292

[139] Kroemer G Reed JC Mitochondrial control of cell death. Nat Med 2000 6 513 9 10802706 10.1038/74994

[140] Barron MJ Johnson MA Andrews RM Clarke MP Griffiths PG Bristow E He LP Durham S Turnbull DM Mitochondrial abnormalities in ageing macular photoreceptors. Invest Ophthalmol Vis Sci 2001 42 3016 22 11687550

[141] Wallace DC A mitochondrial paradigm of metabolic and degenerative diseases, aging, and cancer: a dawn for evolutionary medicine. Annu Rev Genet 2005 39 359 407 16285865 10.1146/annurev.genet.39.110304.095751 PMC2821041

[142] Feher J Kovacs I Artico M Cavallotti C Papale A Balacco Gabrieli C Mitochondrial alterations of retinal pigment epithelium in age-related macular degeneration. Neurobiol Aging 2006 27 983 93 15979212 10.1016/j.neurobiolaging.2005.05.012

[143] Carelli V Ross-Cisneros FN Sadun AA Mitochondrial dysfunction as a cause of optic neuropathies. Prog Retin Eye Res 2004 23 53 89 14766317 10.1016/j.preteyeres.2003.10.003

[144] Beatty S Koh H Phil M Henson D Boulton M The role of oxidative stress in the pathogenesis of age-related macular degeneration. Surv Ophthalmol 2000 45 115 34 11033038 10.1016/s0039-6257(00)00140-5

